# Quantification of thermal impacts across freshwater life stages to improve temperature management for anadromous salmonids

**DOI:** 10.1093/conphys/coac013

**Published:** 2022-04-03

**Authors:** Alyssa M FitzGerald, Benjamin T Martin

**Affiliations:** 1 Institute of Marine Sciences, University of California Santa Cruz, 1156 High St., Santa Cruz, CA 95064, USA; 2 Fisheries Ecology Division, Southwest Fisheries Science Center, National Marine Fisheries Service, National Oceanic and Atmospheric Administration, 110 McCallister Way, Santa Cruz, CA 95060, USA; 3 Department of Theoretical and Computational Ecology, University of Amsterdam, Science Park 904, 1098 XH Amsterdam, The Netherlands

**Keywords:** Central Valley, Chinook salmon, life cycle, life stage, thermally-mediated survival, thermal performance model

## Abstract

Water temperature is the major controlling factor that shapes the physiology, behaviour and, ultimately, survival of aquatic ectotherms. Here we examine temperature effects on the survival of Chinook salmon (*Oncorhynchus tshawytscha*), a species of high economic and conservation importance. We implement a framework to assess how incremental changes in temperature impact survival across populations that is based on thermal performance models for three freshwater life stages of Chinook salmon. These temperature-dependent models were combined with local spatial distribution and phenology data to translate spatial–temporal stream temperature data into maps of life stage-specific physiological performance in space and time. Specifically, we converted temperature-dependent performance (i.e. energy used by pre-spawned adults, mortality of incubating embryos and juvenile growth rate) into a common currency that measures survival in order to compare thermal effects across life stages. Based on temperature data from two abnormally warm and dry years for three managed rivers in the Central Valley, California, temperature-dependent mortality during pre-spawning holding was higher than embryonic mortality or juvenile mortality prior to smolting. However, we found that local phenology and spatial distribution helped to mitigate negative thermal impacts. In a theoretical application, we showed that high temperatures may inhibit successful reintroduction of threatened Central Valley spring-run Chinook salmon to two rivers where they have been extirpated. To increase Chinook salmon population sizes, especially for the threatened and declining spring-run, our results indicate that adults may need more cold-water holding habitat than currently available in order to reduce pre-spawning mortality stemming from high temperatures. To conclude, our framework is an effective way to calculate thermal impacts on multiple salmonid populations and life stages within a river over time, providing local managers the information to minimize negative thermal impacts on salmonid populations, particularly important during years when cold-water resources are scarce.

## Teaser

We developed a framework to assess thermal impacts for anadromous salmonid populations. We used temperature-dependent survival models to calculate the likelihood of survival to the subsequent life stage. Implementing our framework can help maximize survival for these declining populations, particularly important during years when cold-water resources are scarce.

## Introduction

For aquatic ectotherms, temperature is the primary abiotic factor that controls biophysical, biochemical and bioenergetic processes (Fry, 1971), thus influencing spatial distributions (Fourcade *et al.,* 2018), physiological rates, daily and long-term survival and evolutionary trajectory (Brannon *et al.,* 2004). Seasonal patterns in temperature have resulted in corresponding patterns in life history, phenology and spatial distribution for aquatic populations, such that a geographically widespread species may consist of multiple populations with unique thermal environments and thermal adaptations (Chen *et al.,* 2013; Eliason *et al.,* 2011; FitzGerald *et al.,* 2021; Flitcroft *et al.,* 2016; Nadeau *et al.,* 2017; Quinn, 2018; Stitt *et al.,* 2014; Zillig *et al.,* 2021). Sudden shifts in the long-term thermal average can induce conditions for which a population has not experienced. Anthropogenic-mediated alterations—particularly dams and warming stream temperatures—have resulted in exposure to novel temperature regimes for which aquatic populations are not adapted. Although regulated watersheds should be able to mimic natural thermal regimes, they often do not (Willis *et al.,* 2021). The temperature of the water, timing of release, duration of release, amount of water and discharge rate are constantly debated because the thermal requirements for one life stage or species may be harmful to another (e.g. Zarri *et al.,* 2019), interspecific populations may exhibit different adaptations (Eliason *et al.,* 2011; Zillig *et al.,* 2021) and thermally suitable habitat may vary spatially and temporally (Armstrong *et al.,* 2021; FitzGerald *et al.,* 2021). These conditional effects are not well-studied due to their complexity, indicating a need for a spatially and temporally explicit comparative model that can estimate population-specific thermal impacts on sympatric and successive life stages (Crozier *et al.,* 2017; Crozier *et al.,* 2021; Snyder *et al.,* 2019).

Pacific salmonids—economically, culturally and ecologically important species that have experienced severe declines in recent decades (NRC, 1996; NMFS, 2016)—are ideal species to examine comparative thermal impacts among life stages. First, water temperature is a major factor affecting salmonid survival and evolution in freshwater habitats (Isaak *et al.,* 2017a; Poole *et al.,* 2001; Roper and Scarnecchia, 1999; Todd *et al.,* 2008) and salmonids are sensitive to even minor temperature changes (U.S. EPA, 2003). Water temperature is so important that habitat suitability is sometimes defined solely by water temperature (U.S. EPA, 2003). Second, many populations in the continental USA are present in rivers below dams where the water quality is strictly regulated and managed to meet thermal targets. Managers can adjust water temperature by releasing more cool water from the reservoirs upstream of dams or by altering the timing of water releases. The temperature targets for salmonids are based on the United States Environmental Protection Agency (U.S. EPA)—Region 10 Guidance for Pacific Northwest State and Tribal Temperature Water Quality Standards (U.S. EPA, 2003), hereafter EPA criteria. EPA criteria provide binary (i.e. suitable vs. not suitable) temperature thresholds that managers need to meet in order to protect salmonids and other aquatic organisms. Third, life stages and populations can have different thermal requirements (U.S. EPA, 2003; Zillig *et al.,* 2021), and some life stages and populations co-exist. However, the EPA criteria do not provide guidance on how cold-water resources should best be managed to minimize negative impacts on salmonid populations, especially in instances when temperature requirements cannot be met, conditions that may become more frequent in the future (Feldman *et al.,* 2021). Fourth, salmonids are relatively well studied, and several recent studies have described the relationship between temperature and physiological performance for multiple life stages using thermal performance curves (TPCs) (e.g. Martin *et al.,* 2015; Martin *et al.,* 2017; Perry *et al.,* 2015).

**Figure 1:**
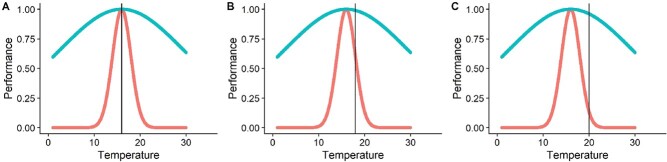
Example showing how altering the temperature (defined by black line) by the same amount can lead to substantially different population impacts if the shapes of the TPCs differ between life stages (stage 1 in pink, stage 2 in teal). In this example, both life stages have the same thermal optimum (temperature at which performance is maximized), but as the temperature shifts away from the optimum (A-B-C), performance decreases slightly for the teal life stage but declines rapidly for the pink life stage.

Here we use TPCs in a spatially and temporally explicit framework to quantify thermal effects across salmonid life stages. TPCs quantify the relationship between temperature and the physiological performance of a particular process (e.g. survival, growth, reproduction). TPCs thus quantify the temperature-dependent effects on a trait, define the optimum temperature (the temperature at which performance is maximized) and describe how deviations from the optimum affect performance (Schulte *et al.,* 2011). If the shape of the TPCs differs substantially among life stages, then exceeding a pre-specified thermal criterion by the same amount can lead to substantially different mortality rates (Fig. 1). TPCs are therefore ideal to compare temperature-dependent responses across life stages, between populations and to changing temperatures (Eliason *et al.,* 2011; Schulte *et al.,* 2011).

**Figure 2:**
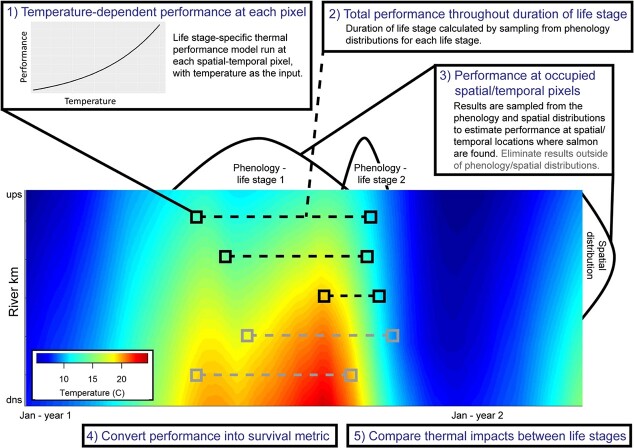
Illustrated framework for assessing thermal impacts of life stages along a river. (1) First, the life stage-specific TPC model is run at each spatial–temporal location, with temperature as the input. (2) Based on known phenology, the total performance throughout the duration of the life stage is calculated. (3) Results are sampled from the phenology and spatial distributions to estimate performance at spatial–temporal locations where salmon are found, as opposed to the whole river throughout the year. (4) To compare life stages, performance is converted into a metric of survival (see text). (5) The above steps are repeated for each life stage, and thermal impacts on different life stages are compared.

We developed a framework to quantify thermal impacts across life stages using TPCs for each life stage, local phenological and spatial data to determine when and where each life stage occurs and water temperature along that river (Fig. 2). First, water temperatures are used as inputs in various life stage-specific TPCs that relate temperature to instantaneous physiological performance (step 1 in Fig. 2). Second, these TPCs are combined with local spatial distribution and phenology data and stream temperature to translate spatial–temporal temperature data into maps of life stage-specific performance in space and time (steps 2 and 3 in Fig. 2). Third, thermal performance was compared between life stages by estimating how effects on physiological rates are translated to reductions in survival (steps 4 and 5 in Fig. 2); this important step helps elucidate which life stages are the most vulnerable to negative thermal impacts. Additionally, we compared thermal survival with and without local fish data (i.e. skipping steps 2 and 3), allowing us to quantify if local behaviours help to mitigate negative thermal impacts. We examined thermal effects for pre-spawn adults, embryos and juveniles using the best TPCs currently available for Chinook salmon (*Oncorhynchus tshawytscha*). This comparative framework using continuous TPCs to quantify temperature-dependent survival across life stages can ultimately help prioritize cold water resources to minimize negative impacts on salmonid populations, especially in years when thermal criteria cannot be met.

## Methods

### Stream selection: Central Valley, California

The Central Valley watershed of California is the southernmost extent of the natural Chinook salmon range, and salmon here inhabit highly modified and managed habitats and are routinely exposed to high stream temperatures and severe and prolonged droughts. Water temperature often meets or exceeds EPA thresholds, especially during years of extreme temperature or drought. For example, in 1977 in the second of two sequential drought years, water temperatures in the Sacramento River exceeded the requirements of every life stage from July through October (Boles, 1988). A 4-year drought from 2012–2016 resulted in high egg mortality of winter- and fall-run when temperatures in the Sacramento River exceeded 16°C (Bland, 2015), above the EPA threshold of 13°C for spawning and incubation (U.S. EPA, 2003). Additionally, the climate projections indicate that Central Valley reservoirs may not be able to meet salmonid thermal criteria or other operational requirements during future droughts (Feldman *et al.,* 2021).

We illustrated our TPC modelling framework on three rivers in the Central Valley: Clear Creek, Stanislaus River and Tuolumne River (Table S1.1); note, however, that our framework can easily be expanded to other rivers depending on data availability. Clear Creek has both fall- and spring-run Chinook salmon. A salmonid population is conventionally named by its natal stream and the seasonal ‘run’ timing of adult freshwater entry and migration, such as Clear Creek fall-run Chinook salmon and Clear Creek spring-run Chinook salmon. Here, adults of the two populations arrive to Clear Creek in different seasons, but spawn timing can partially overlap, and juveniles may rear sympatrically. The Stanislaus and Tuolumne Rivers currently only have fall-run because spring-run have likely been extirpated. A few spring-run like (based on timing only) adults and juveniles are found regularly in the Stanislaus River, but it is unclear if these individuals are hatchery strays or represent a very small breeding population (Franks, 2014). Also note that our work does not examine the recent spring-run reintroductions to the San Joaquin River basin by the San Joaquin River Restoration Program (http://www.restoresjr.net/).

Each river in our study is partitioned by a large dam that blocks anadromous fish passage to formerly accessible high-elevation habitat, but fish still spawn and rear downstream of the dams. These streams also represent different linear amounts of available habitat for salmon: less than 50 km for Clear Creek and 50–100 km for Stanislaus and Tuolumne Rivers. Although the levels of discharge in each river vary considerably within and among years, Clear Creek has historically had the lowest peak discharge, Stanislaus has had the highest peak discharge and the Tuolumne has had a moderate peak discharge (Table S1.1; Fig. S1.1). Critically, each river in our study has well-recorded stream temperature data and salmonid life-history demographics, which are input requirements for our framework. Finally, because each river is regulated by a hydroelectric dam, managers can change the discharge rate, timing of release and water temperature to support aquatic populations.

### Pre-processing: temperature

To identify which life stages will be most severely impacted during years of elevated temperature, we illustrated our models during a period of hot, intense drought in California, years 2013–2014 (Swain *et al.,* 2014, CDWR, 2020). Daily stream temperature was the basic input into each river model. Observed mean daily temperatures were extracted for each river from the temperature monitors used by Isaak *et al.* (2017b) in the NorWest project (downloaded from https://www.fs.fed.us/rm/boise/AWAE/projects/NorWeST/StreamTemperatureDataSummaries.shtml) using ArcMap. To obtain daily stream temperatures for all river kilometres, we linearly interpolated actual stream temperature observations to create stream temperatures along a river that matched the spatial–temporal resolution of the spatial and phenological data (i.e. 1 km/daily). Gaps of less than 30 days were linearly interpolated for all monitors before spatial interpolation. In the rare instance where more than one temperature recording was present per river kilometre for a specific date, we took the average.

### Step 1: TPCs applied to each spatial–temporal pixel

**Figure 3:**
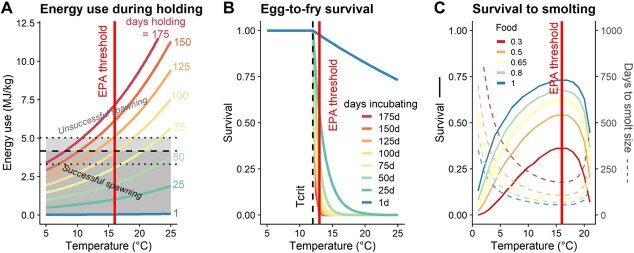
TPCs are applied to each spatial–temporal pixel for each Chinook salmon life stage. Several inputs, particularly temperature, life stage duration and food availability (juveniles only) can significantly impact physiological performance and survival. For reference, we include the EPA thermal thresholds for each life stage (red lines; U.S. EPA, 2003). (**A**) Energy use during pre-spawn holding increases exponentially with temperature and depends on the numbers of days holding before spawning. To spawn successfully, the amount of energy used during holding must be below the dashed line based on average initial energy density (±1 SD, dotted lines, shown here for spring-run). (**B**) Egg-to-fry survival decreases exponentially above T_CRIT_ (12°C, dashed line), the temperature below which no temperature-dependent mortality occurs, and depends on the number of days of incubation before emergence. (**C**) Temperature determines a juvenile’s growth rate. Survival to smolt size (bolded lines, left axis) increases as days to reach smolt size (dotted lines, right axis) decreases. The maximum instantaneous juvenile growth rate is at T_OPT_ = 16°C, which is also the EPA threshold for core juvenile rearing. Food availability significantly impact survival to smolt size. Here, we set *Food* = 0.65 (see text).

We calculated temperature-dependent performance for three life stages: adults during pre-spawning holding, embryos during incubation and juveniles smolting in-stream. Each TPC was run at each spatial–temporal pixel, representing stream temperature across space and time (i.e. 1 km/day), with temperature as the input (Fig. 2). We then quantified performance throughout the duration of each life stage (described in detail below; Fig. 3). Unless otherwise stated, all analyses were completed in R.

#### Model 1: metabolic expenditure during holding

Sexually immature spring-run Chinook salmon adults migrate towards the spawning grounds a few months before spawning is initiated, holding in low-velocity pools during the warmest stream temperatures of the year as their gonads mature (Moyle, 2002). During this period, they do not eat (McCullough, 1999) and therefore have a finite amount of energy to cover the costs of maintenance metabolism. Energy use increases exponentially with temperature, so here we employed a temperature-dependent metabolic expenditure model to calculate the energy expended by an adult holding prior to spawning (from Martin *et al.,* 2015):

\begin{align*} B_{h\,i}=c_{0} M^{b}\ e^{DT}, \end{align*}

where *B_h i_* is the maintenance metabolism, or how much energy is used by an adult salmon at a given temperature per unit time *i* (Fig. 3A). Values for *b* (−0.217 [unitless]), the mass exponent of maintenance, and *D* (0.068 [°C^−1^]), the temperature exponent of maintenance, were originally parameterized by Rao (1968) on rainbow trout and were adapted to Chinook salmon in Lake Michigan by Stewart (1980). The *c_0_* value was parameterized using Chinook salmon data (Martin *et al.,* 2015) and converted to reflect our analyses on a daily time scale (1565.04 mgO_2_ d^−1^ kg^−1^). The mass, *M*, was estimated at 7.37 kg based on the relationship between fork length (*L_F_*) and somatic and gonadal masses at the start of migration (average female *L_F_* = 815 mm; Bowerman *et al.,* 2017). *T* is temperature (°C) for a given location and time obtained from linear interpolations of observed stream temperatures.

After running the model to calculate the maintenance metabolism at a single location in space and time, we summed daily energetic expenditures across time to determine the total energy used per unit mass by an adult holding for *n* time steps (here, days) at a given location:

$$ {E}_{hTOT}=\sum_{i=1}^n{B}_{h\ i}t, $$

where *n* is the number of days holding and *t* represents the time step, in our example, 1 day (Fig. 3A). The length of the holding period, *n*, was determined by the differential between day of arrival and spawning date. Because little information exists to calculate the exact duration of holding for a specific fish, we randomly sampled spawning date from the phenological distribution 1000 times, running the model each time at each spatial–temporal point; the standard deviation of energy expenditure (*E_hTOT_*) was low for all locations in space and time (max, 0.419; Fig. S2.1). We began running our model from the earliest arrival day for each river minus a 30-day buffer; for example, the earliest arrival day on Clear Creek was Julian Day 83 (around March 25) so we began running our model from Julian Day 53 (around February 23). Similarly, we included a 30-day buffer after the last reported spawning date, but salmon arriving after peak spawning were assumed to spawn immediately and have no holding costs. These buffers allowed us to estimate holding costs for early arrivals and late spawners that may be missing from our dataset due to low abundance, lack of sampling or inability to distinguish from other runs. Results were averaged over the 1000 replicates and converted from mgO_2_ kg^−1^ to MJ kg^−1^ (1 mgO_2_ = 1.358442*10^−5^ MJ; see Martin *et al.,* 2015).

#### Model 2: embryonic mortality

The primary physical factors inducing salmonid embryonic mortality are water temperature (very high or very low), fine sediment, low dissolved oxygen, and scour from high flow events (Quinn, 2018). Because embryonic survival and incubation duration are strongly temperature-dependent (McCullough, 1999), and because high water temperatures in the Central Valley can exceed recommended incubation thresholds (U.S. EPA, 2003) during normal water years (Boles, 1988), we applied a temperature-dependent embryo mortality model based on daily incubation temperatures to calculate the percent mortality of eggs spawned at a given location in space and time (Martin *et al.,* 2017):

$$ {M}_T=1-\prod_{i=1}^n\exp \left(-{s}_T\left({T}_i-{T}_{crit}\right)\right), $$

where *M_T_* is the temperature-dependent mortality throughout the embryonic period, *s_T_* is a parameter defining the slope at which the mortality rate increases with temperature above *T_crit_*, *T_crit_* is the temperature below which there is no mortality due to temperature and *T_i_* is the temperature experienced at the *i*th day of development (Fig. 3B). The model was parameterized with winter-run Chinook salmon field observations of egg-to-fry survival data, resulting in *T_crit_* = 12.0°C and *s_T_* = 0.024°C^−1^d^−1^ (Martin *et al.,* 2017).

In the Central Valley, embryos generally incubate for 2–4.5 months prior to emergence, depending on temperature (Boles, 1988). To determine the length of the embryonic period, *n*, at each spatial–temporal location, we implemented the temperature-dependent maturation function by Zeug *et al.* (2012):

$$ 1\le \sum_{i=1}^n0.001044\ {C}^{-1}{d}^{-1}\times {T}_i+0.00056\ {d}^{-1}, $$

where emergence occurs when the sum reaches 1. The temperature-dependent embryo mortality model was then run using the calculated embryonic period for each spatial–temporal location.

#### Model 3: juvenile growth rate

Fish size and exposure temperature can influence a juvenile’s ability to successfully smolt (Ewing *et al.,* 1979; Healey, 1991; McCullough, 1999), such that fish with low growth rates may undergo desmoltification, revert to parr and return to freshwater, resulting in subsequent high juvenile mortality (McCullough, 1999). Growth rates of fish are temperature-dependent, and so we applied a juvenile growth model based on temperature (Perry *et al.,* 2015):

Ω = *d* * (*T* – *T_L_*) * (1 – exp (*g* * (*T* – *T_U_*))),

where Ω is the mass-standardized growth rate (specific growth rate per 1 g of fish); *T* is mean temperature over the growth period; *d* and *g* are shape parameters; and *T_L_* and *T_U_* are parameters defining the lower and upper thermal limits, respectively, at which the growth rate is zero. Perry *et al.* (2015) parameterized the model with experimental juvenile growth data from multiple Chinook salmon populations, resulting in *d* = 0.415, *g* = 0.315, *T_L_* = 1.833°C and *T_U_* = 24.918°C.

Unlike the adult energy expenditure model (Model 1) and the embryonic mortality model (Model 2), which were parameterized from field studies, the above juvenile growth parameters were estimated from juvenile salmonids reared in laboratory conditions that were unrepresentative of conditions in the field (e.g. *ad libitum* rations). These artificial laboratory conditions may have underestimated thermal impacts. For example, Childress and Letcher (2017) found that TPCs for brook and brown trout in the field were reduced by 2–3°C in the field compared to laboratory studies. Martin *et al.* (2017) found a similar shift in thermal tolerance for Chinook salmon winter-run embryos from laboratory to field. Following Childress and Letcher (2017) and Martin *et al.* (2017), we applied a temperature correction factor, *T_corr_* = 3.0°C:

Ω = *d* * (*T* + *T_corr_* – *T_L_*) * (1 – exp (*g* * (*T* + *T_corr_* – *T_U_*))).

Ration level can also significantly impact growth rate (Brett *et al.,* 1969; Brett *et al.,* 1982), so we also included the feeding level *Food*, the fraction of the maximum growth rate due to food limitation (*ad libitum* = 1; Fig. 3C):

Ω = Food*d * (*T* + *T_corr_* – *T_L_*) * (1 – exp (*g* * (*T* + *T_corr_* – *T_U_*))).

Here, we assumed that *Food* = 0.65 based on a study of juvenile Chinook salmon growth in the field relative to the laboratory (Brett, 1982). However, we note that the *Food* parameter likely fluctuates over time and varies between rivers but is understudied in the Central Valley. Finally, the relative growth rate (100 * Ω/Ω_max_) was calculated at spatial and temporal locations.

### Steps 2 and 3: results sampled from phenological and spatial distributions

First, phenological distributions were used to determine the duration of a life stage if the actual duration was unknown; for example, the length of pre-spawning holding was determined by sampling from the arrival and spawning phenological distributions. After calculating thermal performance results along the entire spatial–temporal matrix (i.e. the whole river for the entire time period), we next sampled from the empirical phenological and spatial distributions in order to estimate performance at spatial–temporal locations where and when a life stage is present. For these weighted results, the number of sampling replicates equaled the size of the entire unweighted temperature matrix (i.e. the number of river kilometre multiplied by the number of days).

We obtained phenological and spatial data from published sources, technical reports and/or raw data for each life stage per stream (Table S1.1). We fit a daily phenological distribution model based on Julian day and count (e.g. number of live fish or redds) for each life stage along each river (Supplement 3; Table S3.1; Fig. S3.1). Specifically, we obtained empirical data for adults arriving to the spawning grounds and spawn timing. Similarly, we fit spawning and rearing spatial distribution models based on count per river kilometre from redd surveys and juvenile observations (Supplement 3; Table S3.2; Fig. S3.1). We defined adult holding habitat from redd surveys because holding often occurs on or near the natal grounds (Quinn, 2018; Yoshiyama *et al.,* 2001). Whenever possible, we analysed multiple years of data to obtain the long-term average distribution because phenology and even spatial distribution can vary between years (e.g. Ford and Brown, 2001) and because fish data was not as consistent as temperature data across years. Raw counts were converted into percentages based on the total count for that year to weight all years equally regardless of that year’s population size. We calculated summary statistics and then sampled from the fitted distributions for use in thermal performance models. Unless otherwise stated, Gaussian (normal) models were fit.

### Step 4: performance metrics converted to survival

The models that we implemented calculate different metrics of performance: adult energy expenditure, embryonic mortality and juvenile relative growth rate. To assess thermal effects across life stages, we converted all temperature-dependent performance metrics into the likelihood of survival to the next life stage.

#### Likelihood of successful spawning

**Figure 4:**
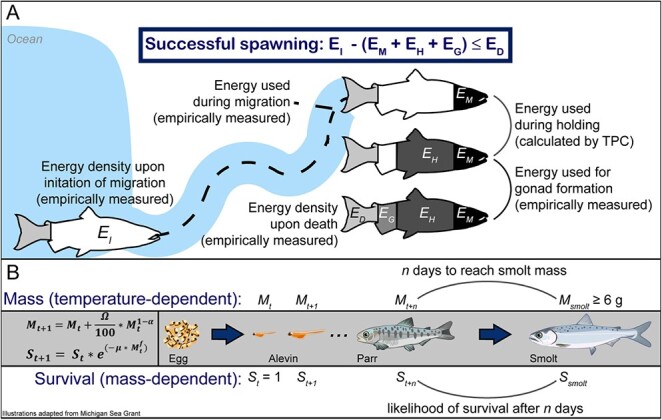
Step 4: Conceptual figures illustrating the conversion of thermal performance metrics into survival. (**A**) Likelihood of successful spawning based on energy expended throughout migration, holding and spawning. (**B**) Likelihood of smolting based on temperature-dependent growth rate and size-dependent predation. See text for details on variables and parameters.

Adult salmon do not consume food during migration or spawning, and therefore have a finite amount of energy once migration commences to reach the spawning grounds, hold until proper spawning conditions, form gonads and spawn. Post-spawning, salmon carcasses have ~ 4 MJ kg^−1^ of energy (*E_D_*), indicating that this energy is unavailable for migration or reproduction costs (Bowerman *et al.,* 2017; Crossin *et al.,* 2004; Plumb, 2018). Therefore, in order to successfully survive to spawn, energy expenditure must not exceed *E_D_:*

$$ {E}_I-\left({E}_M+{E}_H+{E}_G\right)\le {E}_D, $$

where *E_I_* is the energy at the start of migration, *E_M_* is energy used during migration, *E_H_* is energy used during holding and *E_G_* is energy allocated to gonad formation (Fig. 4A). These parameters can differ to some degree both within and among populations (Bowerman *et al.,* 2017; Crossin *et al.,* 2004), so we assumed a variable starting muscle energy density (spring-run: *E_I_* = 11.7 ± 1.0 MJ kg^−1^, Bowerman *et al.,* 2017; fall-run: 8.0 ± 1.0 MJ kg^−1^, Martin *et al.,* 2015). Migration costs depend on the migratory difficulty, i.e. distance travelled, elevation, speed, temperature and flow (Bowerman *et al.,* 2017; Crossin *et al.,* 2004; Martin *et al.,* 2015; Mesa and Magie, 2006). Fall-run Chinook salmon in the Sacramento River expended ~2 MJ kg^−1^ during migration (Martin *et al.,* 2015) whereas spring-run Chinook salmon travelling ~1150 km to the South Fork Salmon River in Idaho expended ~5 MJ kg^−1^ (Bowerman *et al.,* 2017). Energy costs for spring-run Chinook salmon migrating 200–500 km in the Central Valley are likely higher than for fall-run in the same system but less than spring-run that travel twice as far, so we set an energy expenditure of *E_M_* = 2.5 MJ kg^−1^ for spring-run. Our temperature-dependent metabolic expenditure model calculates holding costs (*E_H_*; see above). Gonad formation (*E_G_*) costs 14% of starting somatic energy density, based on a study of migrating spring-run Chinook salmon in the Columbia/Snake/Salmon Rivers (Bowerman *et al.,* 2017), and we assumed that a similar percentage is allocated from muscle. It is unknown if Central Valley runs invest different proportions of their initial energy reserves to gonad formation, so we assumed that fall-run and spring-run invested the same proportion. After death, post-spawned females have an average energy density of 3.4 MJ kg^−1^ (Bowerman *et al.,* 2017), providing an estimate for *E_D_*. We use values for muscle energy density because muscle energy stores decline significantly during holding (Bowerman *et al.,* 2017; Mesa and Magie, 2006).

We randomly sampled from the *E_I_* distribution 1000 times and ran the above model for each spatial–temporal pixel to calculate the percent likelihood that an individual would have enough energy to successfully survive to spawn (i.e. energy used is ≤ *E_D_*).

#### Likelihood of egg-to-fry survival

The performance metric from Model 2 calculated the proportion of embryonic mortality, so we simply translated this metric into egg-to-fry percent survival.

#### Likelihood of successful smolting

To successfully smolt and enter the marine environment, juveniles need to reach a size of approximately 6 g (Ewing *et al.,* 1979; Larsen *et al.,* 2010; Sharron, 2015). Our model focuses on juveniles that rear on the natal grounds until smoltification, meaning that these yearlings need to remain in-stream until they reach this size. We calculated daily mass based on the temperature-dependent growth rate until the smolt size threshold was reached (Fig. 4B):

$$ {M}_{t+1}={M}_t+\frac{\varOmega }{100}\ast {M}_t^{1-\alpha }, $$

where *M_t_* is the previous mass, *M_t + 1_* is the new calculated mass, Ω is the mass-standardized growth rate from above and *α* is the allometric growth constant, calculated as 0.338 for juvenile Chinook salmon (Perry *et al.,* 2015). Based on the relationship between the mass of eggs and emergent fry and an average Chinook salmon egg weight of 0.3 g (Beacham and Murray, 1990; Quinn, 2018), we calculated that Chinook salmon fry emerged on Day 1 with mass 0.46 g (i.e. *M_1_* = 0.46 g).

Juvenile mortality is high in-stream primarily due to predation, and mortality rates are strongly size-dependent, putting immense pressure on small fish to grow quickly and outmigrate (Lorenzen, 1996). The probability of surviving to day *t + 1* is given by:

$$ {S}_{t+1}={S}_t\ast {e}^{-\mu }, $$

where *S_t_* is the previous survival probability and *μ* is the daily mortality rate. *μ* depends on the mass of the fish at time *t* (*M_t_*):

$$ \mu ={X}_u\ast {M}_t^f, $$

where *X_u_* is the background mortality rate per 1 g of fish and *f* is the allometric scaling factor (Lorenzen, 1996; Peterson and Wroblewski, 1984). For salmonids in natural systems, Lorenzen (1996) calculated *X_u_* = 0.00753 d^−1^ (or 2.75 y^−1^) and *f* = −0.27.

**Figure 5:**
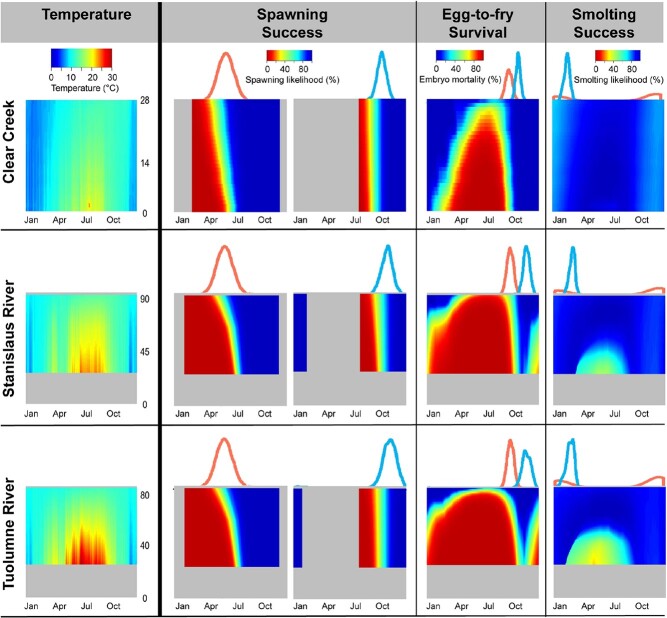
Interpolated temperatures (left panel) and application of temperature-dependent models (right panels) along Clear Creek, Stanislaus River and Tuolumne River for 2013. The horizontal axis represents time, and the vertical axis shows river kilometre number from the confluence (river km 0) upstream to an impassible dam [Clear Creek: Sacramento River upstream to the Whiskeytown Dam (river km 28); Stanislaus River: San Joaquin River upstream to the Goodwin Dam (river km 93); Tuolumne River: San Joaquin River upstream to LaGrange Dam (river km 86); scale for each river is shown on the temperature figure (left panel)]. Left panel: Interpolated temperatures. Temporal gaps of less than 30 days were filled in prior to spatial interpolation. Grey pixels are areas with no data. Right panels: Each location in space and time (pixel) indicates the likelihood of survival of that life stage. We then sampled from fitted phenological distributions (spring-run, pink; fall-run, blue) and spatial distributions (not shown) to weight results. For Spawning Success, we sampled from arrival phenology to calculate the likelihood of adult survival during migration, holding and spawning. For Egg-to-fry Survival, we sampled from spawning phenology to calculate survival during the embryonic period. For Smolting Success, we sampled from emergence phenology to calculate the likelihood of a juvenile to reach smolting size. For the Stanislaus River and Tuolumne River, we applied Clear Creek spring-run phenology to assess potential thermal effects for spring-run on those rivers. Spawning success was run separately for spring-run and fall-run on Clear Creek because the length of the holding period was calculated from arrival date (see text). Grey areas were outside of the temporal buffer zone or had no temperature data and were not run.

The daily mass and survival probability until smolt size was reached was calculated based on daily temperature within the river. In optimum field conditions where juveniles always experience the maximum growth rate (i.e. *T* ~ 16°C), our model predicts that juveniles reach smolt size in 83 days and have a maximum survival of 62%. We present our results as survival at a given thermal exposure as a percent of the maximum survival to smolt size that would occur at optimum temperatures.

### Step 5: thermal effects compared across life stages and scenarios

We first examined the thermal survival results for each life stage along each river using unweighted heatmaps, assuming that each pixel in space and time had an equal likelihood of salmon occupancy (*i.e.* skipping step 3 in Fig. 2). In other words, the unweighted results assumed that salmon phenology and spatial distribution were uniform across space and time, such that life stage decisions (e.g. arrival timing, spawning location, rearing distribution) were not influenced by water temperature. We then compared thermal effects between life stages using weighted results (i.e. including step 3 in Fig. 2) with violin plots, where each result in space and time represents one observation, i.e. one spatial–temporal pixel. Third, we compared weighted and unweighted results to determine if a life stage’s observed phenology and/or spatial distribution helped to mitigate negative thermal effects.

## Results

Mean daily temperature along the three rivers ranged from 5.3°C (Clear Creek in January) to a maximum of 31.6°C (Tuolumne River in July), varying by time of year, spatial location (i.e. upstream or downstream) and river (Fig. 5, left panel). Clear Creek had the coolest temperatures throughout the year, and upstream and downstream locations had similar temperatures. The Stanislaus and Tuolumne Rivers showed somewhat similar thermal profiles, and both of those rivers showed more upstream-to-downstream variation in temperature compared to Clear Creek. Based on thermal profiles alone, we would expect thermal impacts on Chinook salmon to be the least negative along Clear Creek because it is cooler and that the Stanislaus and Tuolumne Rivers would show similar thermal impacts.

**Figure 6:**
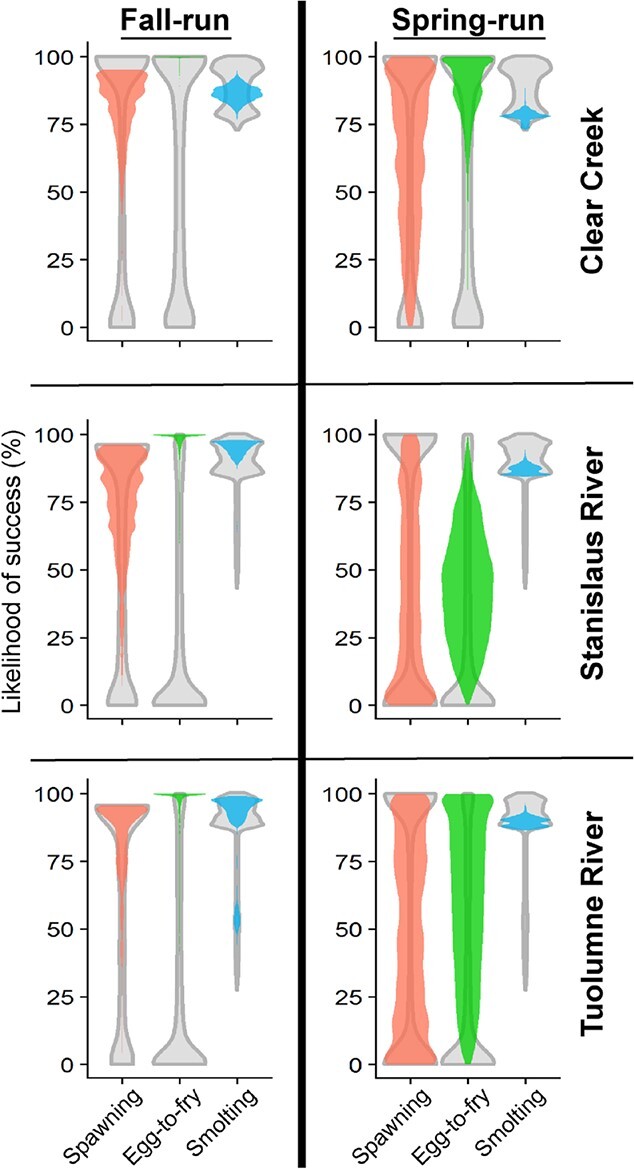
Comparison of thermal effects on different life stages along rivers during 2013. Weighted (coloured) results were based on empirical spatial and phenological distributions whereas unweighted (gray) results assumed that salmon could occur anywhere in the river throughout the year. The likelihood of success (%) represents the likelihood that (1) adults holding will have enough energy to successfully spawn, (2) eggs will successfully emerge as fry and (3) juveniles will experience a high-enough growth rate to smolt. Note that fall-run shows very high weighted egg-to-fry likelihoods (green, left panels) for all three rivers.

We first calculated survival without taking population-specific spatial distribution or phenology into account (Fig. 5, right panels; Fig. 6, grey violin plots). In general, survival was lower downstream and in the summer, when reaches were warmer (Fig. 5, right panels). However, population-specific spatial distribution and phenology influenced thermal effects. For all populations, spatial distribution and phenology helped to mitigate negative thermal impacts (Fig. 6). In other words, a life stage was generally not found in spaces/times where/when negative effects occur. For example, our model calculated that most Clear Creek spring-run fish arriving prior to around mid-May would have no energy to allocate to spawning (Fig. 5, right panels), but peak arrival occurs in early June (Table S3.1; Fig. S3.1). Similarly, Stanislaus River fall-run spawning in the latter half of their season downstream would experience higher embryonic mortality (Fig. 5, right panels), but most spawning occurs upstream (Table S3.2; Fig. S3.1).

A comparison of thermal effects across life stages and runs revealed that fall-run showed higher success for each life stage when compared to spring-run (Fig. 6). The most negatively impacted run/life stage was spring-run adults; spring-run experienced lowered spawning success due to high holding costs from the long period of holding prior to spawning. Egg-to-fry survival was usually >50%, and juveniles experienced relatively high likelihood to reach smolt size for both runs in all rivers.

We ran hypothetical models to assess the potential thermal effects on spring-run populations that have been extirpated along the Stanislaus and Tuolumne Rivers (but see Franks, 2014). For this analysis, we applied the spring-run phenology (i.e. arrival timing, spawn timing, and emergence timing) from Clear Creek, where spring-run are not extirpated, with the fall-run spatial distribution (i.e. redd distribution and rearing distribution) from each river to weight results. Hypothetical spring-runs along the Stanislaus and Tuolumne Rivers would experience high levels of pre-spawning mortality and embryo mortality compared to empirical data from Clear Creek (Fig. 6). Our hypothetical models indicate that most spring-run arriving in these rivers prior to the mean arrival date from Clear Creek would experience detrimentally high temperatures during holding prior to spawning. Similarly, only eggs spawned later in the spring-run spawning season would likely survive. As expected, most juveniles had high predicted likelihood to smolt, potentially even higher than currently experienced in the cooler waters of Clear Creek.

## Discussion

### Summary

We developed a framework to calculate how incremental changes in water temperature impact multiple salmonid populations and life stages within a river over time. Previous studies examining thermal effects of salmonids have often focused on a single life stage, applied behaviour-based simulations in place of empirical information, examined performance rather than survival, or used a coarse spatial–temporal scale; additionally, many of these studies were on Pacific Northwest populations (Crozier *et al.,* 2017; Fullerton *et al.,* 2017; Martin *et al.,* 2017; Perry *et al.,* 2015; Snyder *et al.,* 2019). Here, our thermal survival framework incorporated salmonid empirical phenology and spatial observations, converted thermally mediated physiological performance into estimated impacts on survival, inputted fine-scale spatial–temporal stream temperature data, and compared multiple life stages of multiple California populations simultaneously. Our framework is relatively simple to implement and can be changed, altered, or added to as more data or better models become available.

Calculating effects on different life stages for specific rivers in the Central Valley is particularly timely as it becomes clearer that populations exhibit temperature-dependent differences and that California populations experience higher temperatures than Pacific Northwest populations (FitzGerald *et al.,* 2021; Zillig *et al.,* 2021). Thermal impacts were specific to each Chinook salmon run type, life stage and/or river because of differences in life stage-specific performance, water temperature and local phenology and spatial distribution. Our results therefore indicate that federally defined salmonid thermal thresholds—binary thresholds that ignore sympatric life stages with varying thermal optima—may not maximize a population’s success. Because salmonids are economically important but some populations—especially in California—are declining (Moyle *et al.,* 2017), a management priority should be to limit mortality across life stages. We note, however, that our current framework quantifies temperature-dependent survival of three freshwater life stages rather than population productivity; ideally, our life stage-specific models will be incorporated as inputs into a more comprehensive life cycle model that calculates abundance (e.g. Crozier *et al.,* 2021).

### Empirical thermal impacts on salmonids within and among Central Valley rivers

During a period of abnormally hot temperatures and intense drought in California (Swain *et al.,* 2014, CDWR, 2020), our approach predicted that spring-run Chinook salmon would have lower rates of survival than fall-run, and the most negatively impacted life stage was adults holding prior to spawning. Although pre-spawning mortality data in the Central Valley is sparse, Giovanneti and Brown (2009) found that 78% of female spring-run carcasses had spawned along Clear Creek, agreeing with our predictions that 81% of the spring-run population along Clear Creek would likely survive to spawn. We acknowledge, however, that pre-spawning mortality can vary across years and that water temperature may not be the root cause of mortality for all cases (Bowerman *et al.*, 2017). Still, if temperatures are warmer than average, pre-spawning mortality may be increased, particularly for early arrivals, either directly due to temperature or because of pathogen outbreaks facilitated by warmer temperatures (Ward *et al.,* 2004; Bowerman *et al.*, 2017).

Thermal impacts were strongly influenced by local phenology. Specifically, spring-run Chinook salmon (Clear Creek only) experienced more negative thermal effects than fall-run due to exposure to warm temperatures during the long holding period before spawning, a result that is mirrored by other work (Crozier *et al.,* 2019; FitzGerald *et al.,* 2021). On the Clear Creek spawning grounds, spring-run were present for an average of 112 days longer than fall-run and spawned an average of 31 days earlier; consequently, spring-run experienced detrimentally warm temperatures during the summer and the first half of their spawning season whereas fall-run did not because fall-run enter Clear Creek after thermally intolerable summer temperatures decline to tolerable levels. However, some fall-run fish may have to travel in warmer conditions than spring-run, potentially increasing energy expenditure (Bowerman *et al.,* 2021; Keefer *et al.,* 2018). Our model currently does not quantify how adult migration costs vary for individual fish because there is only one such study in the Central Valley (Martin *et al.,* 2015). In the Central Valley, field research is needed to quantify individual migration costs and migratory difficulty.

Still, local phenology in combination with spatial distribution helped to mitigate negative thermal effects on the spawning grounds and increase resilience; in other words, salmon spawned in locations in space and time that helped to minimize each life stage’s mortality. For example, fall-run incubation in the downstream reaches of the Stanislaus River would experience lower embryonic survival relative to upstream, but spawning does not occur downstream (Peterson *et al.,* 2020, this study). Individual fish select spawning sites based on environmental cues such as water temperature, velocity or substrate (Dudley *et al.*, 2022), honing the spatial distributions and phenology of a population over evolutionary timescales to maximize reproductive success (Chen *et al.,* 2013; Eliason *et al.,* 2011; Flitcroft *et al.,* 2016; Nadeau *et al.,* 2017; Quinn, 2018; Stitt *et al.,* 2014). However, it is important to note that the spawning grounds of all populations that we examined are truncated by dams, such that populations are currently spawning at their upstream limits and cannot move upstream to cooler waters. Therefore, while our results indicate that salmon spawned in locations in space and time that minimized each life stage’s mortality, reproductive success would likely have been even higher in the higher-elevation, cooler waters that were formerly exploited prior to the construction of major rim dams in the 20th century (Beechie *et al.,* 2006; FitzGerald *et al.,* 2021; Lindley *et al.,* 2004; McClure *et al.,* 2008; Moyle *et al.,* 2017; Myers *et al.,* 1998). An additional consideration is that the populations in this study include natural- and hatchery-origin fish (Moyle *et al.,* 2017); hatchery fish may spawn at times or sites that yield lower survival relative to that of locally adapted, natural-origin fish (Austin *et al.,* 2021; Peterson *et al.,* 2020), such that reducing hatchery influence within these populations may increase their survival.

### Assessing theoretical thermal impacts: reintroduction, restoration and climate change

Our approach is ideal for identifying possible sites for reintroductions, evaluating restoration scenarios or predicting future in-river survival with climate change. In this study, we evaluated the reintroduction potential for spring-run Chinook salmon along the Stanislaus and Tuolumne Rivers, rivers where spring-run are currently extirpated or found in non-sustaining numbers (Franks, 2014). These rivers experience warmer temperatures than Clear Creek, particularly during the early spring and late summer, and we estimated that spring-run Chinook salmon would likely experience higher temperature-dependent mortality during holding and incubation than fall-run. However, three scenarios may increase survival for these potential spring-run populations. First, Chinook salmon can shift arrival or spawn timing to track environmental cues (Austin *et al.,* 2021; Peterson *et al.,* 2020; Quinn *et al.,* 2001) such that fish adopting a later arrival and spawning period than Clear Creek spring-run would likely experience lower mortality. Second, salmon will behaviourally thermoregulate to avoid high temperatures and other unsuitable conditions (e.g. Berman and Quinn, 1991; Keefer *et al.,* 2018). The Chinook salmon spawning grounds along these rivers abut dams such that fish cannot move upstream to cooler waters. However, temperature-dependent mortality may be reduced by providing more cool-water habitat downstream of a dam; for example, managers can adjust water temperatures by releasing more cool water at specific times or by changing flow release dates (e.g. Clear Creek Technical Team, 2016, 2017), deeper holding pools could be constructed or shading riparian vegetation could be planted. Third, if survival is projected to be low on the spawning grounds, managers may try to compensate for this loss elsewhere. For example, during dry and critically dry years, juvenile salmonids on the Mokelumne River rearing grounds are trapped and trucked to bypass unsuitable migratory conditions (NMFS, 2014). Another option to compensate for poor projected survival is to reduce ocean take; the number of adult fish captured in the ocean is regulated annually (e.g. https://wildlife.ca.gov/Fishing/Ocean/Regulations/Salmon#commercial). A longer-term solution to increase survival may be reintroductions to formerly accessible habitat upstream of impassable dams (Duda *et al.,* 2021; FitzGerald *et al.,* 2021), as these streams are generally cooler (FitzGerald *et al.,* 2021) and because spring-run have been disproportionately extirpated relative to fall-run due to the loss of this high-elevation habitat (Beechie *et al.,* 2006; Gustafson *et al.,* 2007; McClure *et al.,* 2008).

Theoretical application of this framework can identify areas for habitat restoration and prioritize restoration actions (Jorgensen *et al.,* 2021). For example, our model predicted low egg-to-fry mortality in the downstream reaches of the Stanislaus River between late-October and mid-December, but fall-run do not spawn there. High concentrations of fine sediment and low dissolved oxygen likely prevent successful spawning and incubation in the lower Stanislaus River reaches (Mesick, 2001). Removal of this fine sediment may encourage spawning further downstream because temperature is not a barrier.

Our framework may be applied to estimate future thermal impacts under climate change scenarios. Here, we estimated survival during an intensely hot, drought year when cold-water resources were limited and could not always meet the needs of cold-water salmonids (e.g. USFWS & USBR, 2014, 2015); such conditions are expected to increase in the future (Feldman *et al.,* 2021; FitzGerald *et al.,* 2021; Isaak *et al.,* 2017b; Mantua *et al.,* 2010; Williams *et al.,* 2020). However, quantifying survival in normal or wet water years using empirical phenology and spatial distribution would allow us to determine if cold-water resources can be reserved for future use without negatively impacting salmonid life stages. Banking more water during wet years could help reservoirs meet operational requirements during future droughts. The idea of conserving water during wet years, however, assumes that reservoirs have the appropriate storage capacities. Climate change is expected to convert snowfall to rain in many areas, potentially inundating reservoirs in the spring and forcing them to operate under flood conditions, preventing water banking (Feldman *et al.,* 2021).

### Comparison with EPA salmonid thermal criteria

The models we implemented calculate the temperatures above which negative thermal effects begin to occur for each life stage. In general, these temperatures correspond with the EPA temperature thresholds that were developed to be protective of each salmonid life stage (U.S. EPA, 2003). According to our models, the EPA threshold of 16°C for juveniles during core rearing is ideal, maximizing juvenile growth rates. For Chinook salmon, this threshold could even be relaxed and juveniles would still experience positive growth, although this may not be the case if food is scarce, if warm-water diseases are present or for other salmonid species. The EPA holding threshold of 16°C is protective of the average holding adult, but our models predict that adults holding for longer than 100 days at 16°C will not have enough energy to spawn if they entered freshwater with an average energy density. Our models predict ~15% egg-to-fry survival rate at the EPA threshold of 13°C, whereas 12°C results in no temperature-dependent mortality. However, uncertainty in our models necessitates field testing in Central Valley populations, such that EPA criteria may be appropriate for Chinook salmon in general. Still, the EPA criteria were developed for salmonids in the Pacific Northwest, but California salmonids may have different thermal physiologies adapted to a warmer and more variable thermal regime (Zillig *et al.,* 2021). Additionally, the EPA thermal criteria are binary (i.e. suitable vs. not suitable) and do not allow for survival comparisons among sympatric life stages, populations or species. When the EPA criteria cannot be met (e.g. during severe drought), our framework allows local managers to appropriately prioritize cold water resources to minimize the negative thermal impacts on salmonid populations.

### Uncertainty in models and future improvements

The framework we have implemented includes several different models, each with its own parameters and assumptions (for a detailed description of sources of uncertainty in these models, see FitzGerald *et al.,* 2019). In some cases, these models are not specific to Central Valley Chinook salmon populations due to a current lack of information (Zillig *et al.,* 2021), and parameters were borrowed or estimated from other populations. For example, we assumed that adult migration costs for spring-run are higher than for fall-run in the Central Valley and lower than for spring-run migrating along the Columbia/Snake/Salmon Rivers, but we do not have empirical data for migrating Central Valley spring-run. Similarly, gonad mass increases with freshwater entry date (Hearsey and Kinzinger, 2015), indicating that spring-run and fall-run invest different amounts of energy to gonad development during migration; however, it is unknown if Central Valley runs invest different proportions of their initial energy reserves to gonad formation. To ensure that these temperature-dependent survival estimates are accurate for Central Valley populations, we need more work in two areas. First, our models are based off of the current best-available estimates of thermal performance for Chinook salmon, but some of these studies come from populations outside of the Central Valley. Over time, natural selection tunes a population’s thermal physiology to maximize performance in the environmental conditions to which they are exposed, as shown by studies on brook trout (*Salvelinus fontinalis*; Stitt *et al.,* 2014) and sockeye salmon (*Oncorhynchus nerka*; Eliason *et al.,* 2011, Chen *et al.,* 2013); in other words, a population is locally adapted to the long-time environmental average (Quinn, 2018). Thermal performance, therefore, is also likely population-specific. Conducting experiments with Central Valley populations to estimate thermal performance would determine how parameters from other populations apply to Central Valley salmonids and allow for the incorporation of local adaptation into our model predictions.

Second, some of our models are based on laboratory experiments, and field testing of the models is needed to make sure the models are accurately estimating thermal impacts in the wild. The strength of a laboratory experimental approach is the ability to determine the effects of varying a single variable, i.e. temperature, while keeping all other variables (e.g. flow, photoperiod, habitat) constant, usually in the absence of biotic interference. However, laboratory conditions are often not representative of field conditions (e.g. food *ad libitum*), and so can overestimate thermal performance (Brett *et al.,* 1969; Brett *et al.,* 1982; Childress and Letcher, 2017; Martin *et al.,* 2017).

Another consideration is that we examined smolt survival only on the natal grounds. In the Central Valley, smolting on the natal grounds may sustain a population through drought years (Cordoleani *et al.,* 2021), making our work on smolt success particularly important. However, juveniles that leave the natal grounds prior to smolting (i.e. as fry or parr) to rear and smolt in warmer waters also contribute to salmon populations. Beyond size, smolt likelihood and survival may be influenced by factors that facilitate smolting (e.g. photoperiod and recent growth rate; Ewing *et al.,* 1979), inhibit smolting (e.g. high temperatures; McCullough, 1999) or impact predation rates (e.g. predator species; Nobriga *et al.,* 2021), factors that will vary by rearing strategy. Increasing survival for all juvenile rearing strategies would help sustain populations via the portfolio effect (Sturrock *et al.*, 2020; Cordoleani *et al.,* 2021). We did not examine temperature-dependent survival of juveniles during outmigration, when survival rates are very low (Michel, 2019). Although survival during juvenile outmigration decreases as temperature increases (Michel, 2019), the precise temperature-dependent mechanisms impacting survival (e.g. juvenile metabolism, disease, predation rates) are not clear (Nobriga *et al.,* 2021). Future work should quantify survival for other rearing strategies as well as during juvenile outmigration, although we note the difficulty of defining the spatial–temporal movements of juveniles as well as identifying specific runs and populations from juveniles.

Finer-resolution spatial–temporal fish data and stream temperature could help improve our model outputs by accounting more directly for fish behaviour and movement and thermal exposure. For example, here we assumed that pre-spawn holding occurred on the natal grounds (FitzGerald *et al.,* 2021; Quinn, 2018; Yoshiyama *et al.,* 2001), but holding in thermally stratified pools that are cooler than the surrounding river would result in less energy expended during holding and increased survival relative to our predictions. Finer-resolution spatial stream temperature may better resolve small thermal refugia that fish occupy (Fullerton *et al.,* 2018). Similarly, finer-resolution temporal stream temperature that includes daily fluctuations may better quantify acute mortality from high temperatures (Steel *et al.,* 2017). Still, fish may be able to move to avoid daily high temperatures (e.g. Berman and Quinn, 1991; Keefer *et al.,* 2018). A behaviour-based simulation function assuming that fish maximize their fitness every day (e.g. by minimizing energy expenditure during pre-spawn holding by moving to the coldest part of the river each day) would produce a ‘best-case’ scenario of survival (Fullerton *et al.,* 2017; Snyder *et al.,* 2019), which could be contrasted with empirical mortality rates, if available.

## Funding

This work was supported by the California Regional Water Quality Control Board [agreement #16-048–150].

## Data Availability

The R code and stream temperature data needed to replicate our thermal effects results are openly available in the DRYAD data repository at https://datadryad.org/stash/share/RSkJONoGJEJcMFSMX2YBln90UP-sSvm21v36_PGnmYI (private link for sharing prior to publication; public link will be updated upon acceptance). The summary statistics used to create phenology and spatial distributions for each life stage can be found in the supplementary materials of this article.

## Supplementary Material

supplementary_coac013

## References

[ref1] Armstrong JB , Fullerton AH, Jordan CE, Ebersole JL, Bellmore JR, Arismendi I, Penaluna BE, Reeves GH (2021) The importance of warm habitat to the growth regime of cold-water fishes. Nat Clim Chang 11: 354–361. 35475125 10.1038/s41558-021-00994-y PMC9037341

[ref2] Austin CS , Essington TE, Quinn TP (2021) In a warming river, natural-origin Chinook salmon spawn later but hatchery-origin conspecifics do not. Can J Fish Aquat Sci 78: 68–77.

[ref3] Beacham TD , Murray CB (1990) Temperature, egg size, and development of embryos and alevins of five species of Pacific salmon: a comparative analysis. Trans Am Fish Soc 119: 927–945.

[ref4] Beechie TJ , Ruckelshaus M, Buhle E, Fullerton A, Holsinger L (2006) Hydrologic regime and the conservation of salmon life history diversity. Biol Conserv 130: 560–572.

[ref5] Berman CH , Quinn TP (1991) Behavioural thermoregulation and homing by spring Chinook salmon, *Oncorhynchus tshawytscha* (Walbaum), in the Yakima River. J Fish Biol 39: 301–312.

[ref6] Bland A (2015). For California Salmon, Drought And Warm Water Mean Trouble. YaleEnvironment360. Yale School of Forestry & Environmental Studies. https://e360.yale.edu/features/for_california_salmon_drought_and_warm_water_mean_trouble?utm_source=folwd.com.

[ref7] Boles GL (1988) Water temperature effects on Chinook salmon (*Oncorhynchus tshawytscha*) with emphasis on the Sacramento River: a literature review. California Department of Water Resources, Northern District.

[ref8] Bowerman T , Roumasset A, Keefer ML, Sharpe CS, Caudill CC (2018) Prespawn mortality of female Chinook Salmon increases with water temperature and percent hatchery origin. Trans Am Fish Soc 147: 31–42.

[ref9] Bowerman TE , Keefer ML, Caudill CC (2021) Elevated stream temperature, origin, and individual size influence Chinook salmon prespawn mortality across the Columbia River Basin. Fish Res 237: 1–15.

[ref10] Bowerman TE , Pinson-Dumm A, Peery CA, Caudill CC (2017) Reproductive energy expenditure and changes in body morphology for a population of Chinook salmon *Oncorhynchus tshawytscha* with a long distance migration. J Fish Biol 90: 1960–1979. 28211057 10.1111/jfb.13274

[ref11] Brannon EL , Powell MS, Quinn TP, Talbot A (2004) Population structure of Columbia River Basin Chinook salmon and steelhead trout. Rev Fish Sci 12: 99–232.

[ref12] Brett JR , Clarke WC, Shelbourn, JE (1982) Experiments on thermal requirements for growth and food conversion efficiency of juvenile Chinook salmon *Onchorhynchus tshawytscha*. Canadian Technical Report of Fisheries and Aquatic Sciences, No. 1127. Department of Fisheries and Oceans, Fisheries Research Branch, Pacific Biological Station, Nanaimo, BC.

[ref13] Brett JR , Shelbourn JE, Shoop CT (1969) Growth rate and body composition of fingerling sockeye salmon, *Oncorhynchus nerka*, in relation to temperature and ration size. J Fish Res Bd Can 26: 2363–2394.

[ref14] California Department of Water Resources [CDWR] (2020) California's Most Significant Droughts: Comparing Historical and Recent Conditions. State of California.

[ref15] Chen Z , Anttila K, Wu J, Whitney CK, Hinch SG, Farrell AP (2013) Optimum and maximum temperatures of sockeye salmon (*Oncorhynchus nerka*) populations hatched at different temperatures. Can J Zool 91: 265–274.

[ref16] Childress ES , Letcher BH (2017) Estimating thermal performance curves from repeated field observations. Ecology 98: 1377–1387. 28273358 10.1002/ecy.1801

[ref17] Clear Creek Technical Team (2016) Annual Report for the Coordinated Long-Term Operation Biological Opinion. Clear Creek Restoration Program, Central Valley Project Improvement Act.

[ref18] Clear Creek Technical Team (2017) Annual Report for the Coordinated Long-Term Operation Biological Opinion. Clear Creek Restoration Program, Central Valley Project Improvement Act.

[ref19] Cordoleani F , Phillis C, Sturrock A, FitzGerald AM, Whitman G, Malkassian A, Weber PK, Johnson RC (2021) Threatened salmon rely on a rare life history strategy in a modified and warming landscape. Nat Clim Chang 11: 982–988.

[ref20] Crossin GT , Hinch SG, Farrell AP, Higgs DA, Lotto AG, Oakes JD, Healey MC (2004) Energetics and morphology of sockeye salmon: effects of upriver migratory distance and elevation. J Fish Biol 65: 788–810.

[ref21] Crozier LG , Bowerman TE, Burke BJ, Keefer ML, Caudill CC (2017) High-stakes steeplechase: a behavior-based model to predict individual travel times through diverse migration segments. Ecosphere 8: 1–25.29552374

[ref22] Crozier LG , Burke BJ, Chasco BE, Widener DL, Zabel RW (2021) Climate change threatens Chinook salmon throughout their life cycle. Commun Biol 4: 1–14. 33603119 10.1038/s42003-021-01734-w PMC7892847

[ref23] Crozier LG , McClure MM, Beechie T, Bograd SJ, Boughton DA, Carr M, Willis-Norton E (2019) Climate vulnerability assessment for Pacific salmon and steelhead in the California Current large marine ecosystem. PLoS One 14: e0217711. 31339895 10.1371/journal.pone.0217711 PMC6655584

[ref24] Duda JJ , Torgersen CE, Brenkman SJ, Peters RJ, Sutton KT, Connor HA, Pess GR (2021) Reconnecting the Elwha River: spatial patterns of fish response to dam removal. Front Ecol Evol 9: 1–17.

[ref25] Dudley PN , John SN, Daniels ME, Danner EM (2022) Using decades of spawning data and hydraulic models to construct a temperature-dependent resource selection function for management of an endangered salmonid. Can J Fish Aquat Sci 79: 73–81. 10.1139/cjfas-2021-0022.

[ref26] Eliason EJ , Clark TD, Hague MJ, Hanson LM, Gallagher ZS, Jeffries KM, Farrell AP (2011) Differences in thermal tolerance among sockeye salmon populations. Science 332: 109–112. 21454790 10.1126/science.1199158

[ref27] Ewing RD , Johnson SL, Pribble HJ, Lichatowich JA (1979) Temperature and photoperiod effects on gill (Na+ K)–ATPase activity in Chinook salmon (*Oncorhynchus tshawytscha*). J Fish Res Bd Can 36: 1347–1353.

[ref28] Feldman DR , Tadić JM, Arnold W, Schwarz A (2021) Establishing a range of extreme precipitation estimates in California for planning in the face of climate change. J Water Resour Plan Manag 147: 04021056.

[ref29] FitzGerald AM , John SN, Apgar TM, Mantua NJ, Martin BT (2021) Quantifying thermal exposure for migratory riverine species: phenology of Chinook salmon populations predicts thermal stress. Glob Chang Biol 27: 536–549. 33216441 10.1111/gcb.15450

[ref30] FitzGerald AM , John SN, Apgar TM, Martin BT (2019) Quantifying thermal exposures and effects for Central Valley anadromous salmonids. In California Regional Water Quality Control Board. University of California, Santa Cruz Agreement, p. #16-048-150.

[ref31] Flitcroft RL , Lewis SL, Arismendi I, LovellFord R, Santelmann MV, Safeeq M, Grant G (2016) Linking hydroclimate to fish phenology and habitat use with ichthyographs. PLoS One 11: 1–12. 10.1371/journal.pone.0168831 PMC5179265 28006825

[ref32] Ford T , Brown LR (2001) Distribution and abundance of Chinook salmon and resident fishes of the lower Tuolumne River, California. Fish Bull 179: 253–304.

[ref33] Fourcade Y , Besnard AG, Secondi J (2018) Paintings predict the distribution of species, or the challenge of selecting environmental predictors and evaluation statistics. Glob Ecol Biogeogr 27: 245–256.

[ref34] Franks S (2014) Possibility of natural producing spring-run Chinook salmon in the Stanislaus and Tuolumne rivers. National Oceanic Atmospheric Administration (unpublished work).

[ref35] Fry FEJ (1971) The effect of environmental factors on the physiology of fish. In WS Hoar, DJ Randall, eds, Environmental Relations and Behavior. Elsevier, Academic Press, pp. 1–98

[ref36] Fullerton AH , Burke BJ, Lawler JJ, Torgersen CE, Ebersole JL, Leibowitz SG (2017) Simulated juvenile salmon growth and phenology respond to altered thermal regimes and stream network shape. Ecosphere 8: 1–23. 29552374 10.1002/ecs2.2052 PMC5854398

[ref37] Fullerton AH , Torgersen CE, Lawler JJ, Steel EA, Ebersole JL, Lee SY (2018) Longitudinal thermal heterogeneity in rivers and refugia for coldwater species: effects of scale and climate change. Aquat Sci 80: 1–15. 29556118 10.1007/s00027-017-0557-9 PMC5854952

[ref38] Giovannetti SL , Brown, MR (2009) Adult spring Chinook salmon monitoring in Clear Creek, California. 2008 annual report, U.S. Fish and Wildlife Service, Red Bluff Fish and Wildlife Office, Red Bluff, California.

[ref39] Gustafson RG , Waples RS, Myers JM, Weitkamp LA, Bryant GJ, Johnson OW, Hard JJ (2007) Pacific salmon extinctions: quantifying lost and remaining diversity. Conserv Biol 21: 1009–1020. 17650251 10.1111/j.1523-1739.2007.00693.x

[ref40] Healey MC (1991) Life history of Chinook salmon (*Oncorhynchus tshawytscha*). In C. Groot and L. Margolis, eds, Pacific Salmon Life Histories. University of British Columbia Press, Vancouver, BC, pp. 311–394.

[ref41] Hearsey JW , Kinziger AP (2015) Diversity in sympatric Chinook salmon runs: timing, relative fat content and maturation. Environ Biol Fish 98: 413–423.

[ref42] Isaak DJ , Wenger SJ, Peterson EE, Ver Hoef JM, Nagel DE, Luce CH, Chandler GL (2017b) The NorWeST summer stream temperature model and scenarios for the western US: a crowd-sourced database and new geospatial tools foster a user community and predict broad climate warming of rivers and streams. Water Resour Res 53: 9181–9205.

[ref43] Isaak DJ , Wenger SJ, Young MK (2017a) Big biology meets microclimatology: defining thermal niches of ectotherms at landscape scales for conservation planning. Ecol Appl 27: 977–990. 28083949 10.1002/eap.1501

[ref44] Jorgensen JC , Nicol C, Fogel C, Beechie TJ (2021) Identifying the potential of anadromous salmonid habitat restoration with life cycle models. PLoS One 16: 1–22. 10.1371/journal.pone.0256792 PMC8428657 34499669

[ref45] Keefer ML , Clabough TS, Jepson MA, Johnson EL, Peery CA, Caudill CC (2018) Thermal exposure of adult Chinook salmon and steelhead: diverse behavioral strategies in a large and warming river system. PLoS One 13: 1–29. 10.1371/journal.pone.0204274 PMC6150539 30240404

[ref46] Larsen DA , Beckman BR, Cooper KA (2010) Examining the conflict between smolting and precocious male maturation in spring (stream-type) Chinook Salmon. Trans Am Fish Soc 139: 564–578.

[ref47] Lindley SR , Schick R, May BP, Anderson JJ, Greene S, Hanson C, Low A, McEwan D, MacFarlane RB, Swanson C et al. (2004) Population structure of threatened and endangered Chinook salmon ESUs in California’s Central Valley basin. NOAA Technical Memorandum NOAA-TM-NMFS-SWFSC-360.

[ref48] Lorenzen K (1996) The relationship between body weight and natural mortality in juvenile and adult fish: a comparison of natural ecosystems and aquaculture. J Fish Biol 49: 627–642.

[ref49] Mantua N , Tohver I, Hamlet A (2010) Climate change impacts on streamflow extremes and summertime stream temperature and their possible consequences for freshwater salmon habitat in Washington State. Climatic Change 102: 187–223.

[ref50] Martin BT , Nisbet RM, Pike A, Michel CJ, Danner EM (2015) Sport science for salmon and other species: ecological consequences of metabolic power constraints. Ecol Lett 18: 535–544. 25858695 10.1111/ele.12433

[ref51] Martin BT , Pike A, John SN, Hamda N, Roberts J, Lindley ST, Danner EM (2017) Phenomenological vs. biophysical models of thermal stress in aquatic eggs. Ecol Lett 20: 50–59. 27891770 10.1111/ele.12705

[ref52] McClure MM , Carlson SM, Beechie TJ, Pess GR, Jorgensen JC, Sogard SM, Sultan SE, Holzer DM, Travis J, Sanderson BL et al. (2008) Evolutionary consequences of habitat loss for Pacific anadromous salmonids. Evol Appl 1: 300–318. 25567633 10.1111/j.1752-4571.2008.00030.x PMC3352431

[ref53] McCullough DA (1999) A review and synthesis of effects of alterations to the water temperature regime on freshwater life stages of salmonids, with special reference to Chinook salmon. U.S. Environmental Protection Agency, Region 10: 1–291.

[ref54] Mesa MG , Magie CD (2006) Evaluation of energy expenditure in adult spring Chinook salmon migrating upstream in the Columbia River Basin: an assessment based on sequential proximate analysis. River Res Appl 22: 1085–1095.

[ref55] Mesick C (2001) Studies of spawning habitat for fall run Chinook salmon in the Stanislaus River between Goodwin Dam and Riverbank from 1994 to 1997. Fish Bull 179: 217–252.

[ref56] Michel CJ (2019) Decoupling outmigration from marine survival indicates outsized influence of streamflow on cohort success for California’s Chinook salmon populations. Can J Fish Aquat Sci 76: 1398–1410.

[ref57] Moyle P , Lusardi R, Samuel P, Katz J (2017) State of the salmonids: Status of California’s emblematic fishes 2017. University of California, Davis and California Trout, San Francisco, CA, Center for Watershed Sciences, p. 579.

[ref58] Moyle PB (2002) Salmon and Trout, Salmonidae–Chinook Salmon (*Oncorhynchus tshawytscha*) in Inland Fishes of California. University of California Press, Los Angeles, California, pp. 251–263.

[ref59] Myers JM , Kope RG, Bryant GJ, Teel D, Lierheimer LJ, Wainwright TC, Waples RS (1998) Status review of Chinook salmon from Washington, Idaho, Oregon, and California (pp. 443). NOAA Technical Memorandum NMFS-NWFSC-35.

[ref60] Nadeau CP , Urban MC, Bridle JR (2017) Climates past, present, and yet-to-come shape climate change vulnerabilities. Trends Ecol Evol 32: 786–800. 28844791 10.1016/j.tree.2017.07.012

[ref61] National Marine Fisheries Service [NMFS] (2014) Recovery Plan for the Evolutionarily Significant Units of Sacramento River Winter-Run Chinook Salmon and Central Valley Spring-Run Chinook Salmon and the Distinct Population Segment of California Central Valley Steelhead. National Marine Fisheries Service, West Coast Region, California Central Valley Area Office.

[ref62] National Marine Fisheries Service [NMFS] (2016) Final coastal multispecies recovery plan. National Marine Fisheries Service, West Coast Region.

[ref63] National Research Council [NRC] (1996) Upstream: salmon and society in the Pacific Northwest. National Academies Press.

[ref64] Nobriga ML , Michel CJ, Johnson RC, Wikert JD (2021) Coldwater fish in a warm water world: implications for predation of salmon smolts during estuary transit. Ecol Evol 11: 10381–10395. 34367582 10.1002/ece3.7840 PMC8328468

[ref65] Perry RW , Plumb JM, Huntington W, C. (2015) Using a laboratory-based growth model to estimate mass-and temperature-dependent growth parameters across populations of juvenile Chinook Salmon. Trans Am Fish Soc 144: 331–336.

[ref66] Peterson I , Wroblewski JS (1984) Mortality rate of fishes in the pelagic ecosystem. Can J Fish Aquat Sci 41: 1117–1120.

[ref67] Peterson ML , Lee DJ, Montgomery J, Hellmair M, Fuller A, Demko D (2020) Stability in reproductive timing and habitat usage of Chinook salmon across six years of varying environmental conditions and abundance. Fish Manag Ecol 27: 399–416.

[ref68] Plumb JM (2018) A bioenergetics evaluation of temperature-dependent selection for the spawning phenology by Snake River fall Chinook salmon. Ecol Evol 8: 9633–9645. 30386563 10.1002/ece3.4353 PMC6202718

[ref69] Poole G , Dunham J, Hicks M, Keenan D, Lockwood J, Materna E, Spaulding S (2001) Scientific issues relating to temperature criteria for salmon, trout, char native to the Pacific Northwest. Environmental Policy Agency.

[ref70] Quinn TP (2018) The Behavior and Ecology of Pacific Salmon and Trout, 2nd. University of Washington Press, Seattle, WA.

[ref71] Quinn TP , Kinnison MT, Unwin MJ (2001) Evolution of Chinook salmon (*Oncorhynchus tshawytscha*) populations in New Zealand: pattern, rate, and process. Genetica 112/113: 493–513. 11838785

[ref72] Rao GMM (1968) Oxygen consumption of rainbow trout (*Salmo gairdneri*) in relation to activity and salinity. Can J Zool 46: 781–786. 5724487 10.1139/z68-108

[ref73] Roper BB , Scarnecchia DL (1999) Emigration of age-0 Chinook salmon (*Oncorhynchus tshawytscha*) smolts from the upper South Umpqua River basin, Oregon, USA. Can J Fish Aquat Sci 56: 939–946.

[ref74] Schulte PM , Healy TM, Fangue NA (2011) Thermal performance curves, phenotypic plasticity, and the time scales of temperature exposure. Integr Comp Biol 51: 691–702. 21841184 10.1093/icb/icr097

[ref75] Sharron S (2015) Fish Out of Salt Water: Smoltification in Subyearling Chinook salmon from the Laurentian Great Lakes. Electronic Thesis and Dissertation Repository, Paper 2715. University of Western Ontario.

[ref76] Snyder MN , Schumaker NH, Ebersole JL, Dunham JB, Comeleo RL, Keefer ML, Keenan D (2019) Individual based modeling of fish migration in a 2-D river system: model description and case study. Landsc Ecol 34: 737–754. 33424124 10.1007/s10980-019-00804-z PMC7788051

[ref77] Steel EA , Beechie TJ, Torgersen CE, Fullerton AH (2017) Envisioning, quantifying, and managing thermal regimes on river networks. Bioscience 67: 506–522.

[ref78] Stewart DJ (1980) Salmonid predators and their forage base in Lake Michigan: a bioenergetics-modeling synthesis. University of Wisconsin-Madison.

[ref79] Stitt BC , Burness G, Burgomaster KA, Currie S, McDermid JL, Wilson CC (2014) Intraspecific variation in thermal tolerance and acclimation capacity in brook trout (*Salvelinus fontinalis*): physiological implications for climate change. Physiol Biochem Zool 87: 15–29. 24457918 10.1086/675259

[ref80] Sturrock AM , Carlson SM, Wikert JD, Heyne T, Nusslé S, Merz JE, Sturrock HJW, Johnson RC (2020) Unnatural selection of salmon life histories in a modified riverscape. Glob Chang Biol 26: 1235–1247. 31789453 10.1111/gcb.14896 PMC7277499

[ref81] Swain DL , Tsiang M, Haugen M, Singh D, Charland A, Rajaratnam B, Diffenbaugh NS (2014) The extraordinary California drought of 2013/2014: character, context, and the role of climate change. In SC Herring, MP Hoerling, TC Peterson, PA Stott, eds, Bulletin of the American Meteorological Society, 95, S3.

[ref82] Todd AS , Coleman MA, Konowal AM, May MK, Johnson S, Vieira NK, Saunders JF (2008) Development of new water temperature criteria to protect Colorado's fisheries. Fisheries 33: 433–443.

[ref83] U.S. Environmental Protection Agency [U.S. EPA] (2003) EPA Region 10 Guidance for Pacific Northwest State and Tribal Temperature Water Quality Standards, EPA 910-B-03-002.

[ref84] U.S. Fish and Wildlife Service & U.S. Bureau of Reclamation [USFWS & USBR] (2014) Water Year 2013 Final Accounting Fishery and Water Quality Control Plan Actions. Central Valley Operations, USBR California-Great Basin. https://www.usbr.gov/mp/cvo/index.html.

[ref85] U.S. Fish and Wildlife Service & U.S. Bureau of Reclamation [USFWS & USBR] (2015) Water Year 2014 Final Accounting Fishery and Water Quality Control Plan Actions. USBR California-Great Basin, Central Valley Operations. https://www.usbr.gov/mp/cvo/index.html.

[ref86] Ward PD , McReynolds TR, Garman CE (2004) Butte Creek spring-run Chinook salmon, Oncorhynchus tshawytscha: pre-spawn mortality evaluation 2003. In Inland Fisheries Administrative Report (No. 2004-5). California Department of Fish and Game, Chico, California.

[ref87] Williams AP , Cook ER, Smerdon JE, Cook BI, Abatzoglou JT, Bolles K, Baek SH, Badger AM, Livneh B (2020) Large contribution from anthropogenic warming to an emerging North American megadrought. Science 368: 314–318. 10.1126/science.aaz9600. 32299953

[ref88] Willis AD , Peek RA, Rypel AL (2021) Classifying California’s stream thermal regimes for cold-water conservation. PLoS One 16: 1–19. 10.1371/journal.pone.0256286 PMC8378740 34415917

[ref89] Yoshiyama RM , Gerstung ER, Fisher FW, Moyle PB (2001) Historical and present distribution of Chinook salmon in the Central Valley drainage of California. Fish Bull 179: 71–176.

[ref90] Zarri LJ , Danner EM, Daniels ME, Palkovacs EP (2019) Managing hydropower dam releases for water users and imperiled fishes with contrasting thermal habitat requirements. J Appl Ecol 56: 2423–2430.

[ref91] Zeug SC , Bergman PS, Cavallo BJ, Jones KS (2012) Application of a life cycle simulation model to evaluate impacts of water management and conservation actions on an endangered population of Chinook salmon. Environ Model Assess 17: 455–467.

[ref92] Zillig KW , Lusardi RA, Moyle PB, Fangue NA (2021) One size does not fit all: variation in thermal eco-physiology among Pacific salmonids. Rev Fish Biol Fish 31: 95–114.

