# Supplementary material for: Quantification of thermal impacts across freshwater life stages to improve temperature management for anadromous salmonids
Source: Conserv Physiol. 2022 Apr 3;10(1):coac013. doi: 10.1093/conphys/coac013 (PMC9041423; doi:10.1093/conphys/coac013)
Supplement: supplementary_coac013 [file supplementary_coac013.pdf]

SUPPLEMENTAL MATERIALS

FitzGerald, A. M., & Martin, B. T. Quantification of thermal impacts across freshwater life stages to improve temperature management for anadromous salmonids.

Corresponding author email: Alyssa.fitzgerald@noaa.gov

Supplement 1 – Description of streams

Table S1.1. Description of streams in the Central Valley selected to illustrate our framework, and spatial/phenological sources. Temperature monitors are from NorWest. Years are the years with available temperature data. Length indicates the number of km examined, usually to an impassible fish barrier.

	Clear Creek	Stanislaus River	Tuolumne River
Spring-run	Yes	Extirpated^	Extirpated^
Fall-run	Yes	Yes	Yes
Late-fall-run	Yes*	Maybe*	Maybe*
Winter-run	Extirpated	No	No
Temp monitors (n)	14	7	28
Years	2002-2015	1993-2014	1993-2014
Length (km)	29	93	86
Peak historical discharge (ft^3/s)†	~150	~1,500	~850
Spring-run arrivals	Video/photo monitor[9]	NA	NA
Fall-run arrivals	Video/photo monitor[3,4,5,6,7]	Video/photo monitor[11]	Video/photo monitor[11]
Spring-run spawning	Redd counts[8]	NA	NA
Fall-run spawning	Redd counts[10]	Redd counts[11]	Redd counts[12,13,14,15,16]
Juvenile rearing	Verbal[1], Rotary screw trap[2]		

^but see Franks (2014) and San Joaquin River Restoration Program (http://www.restoresjr.net/)
*low numbers and not well-documented
†see Fig. S1.1 for discharge data
[1]Clear Creek Technical Team 2016, [2]Earley et al. 2013, [3]Killam and Johnson 2013, [4]Killam et al. 2014, [5]Killam et al. 2015, [6]Killam et al. 2016, [7]Killam et al. 2017, [8]S. Provins *unpub.*, [9]Clear Creek Technical Team 2017, [10]Meneks 2017, [11]Tsao and Murphey, pers. comm., 2018, [12]Blakeman 2005, [13]Blakeman 2006, [14]Blakeman 2008, [15]O'Brien 2009, [16]FISHBIO 2013

Figure S1.1. Daily discharge (ft^3/s) from 2010-2016 (blue) and median historical daily discharge (orange). Data output graphs from USGS (https://nwis.waterdata.usgs.gov/ca/nwis/uv?), retrieved 25-Jan-2022.

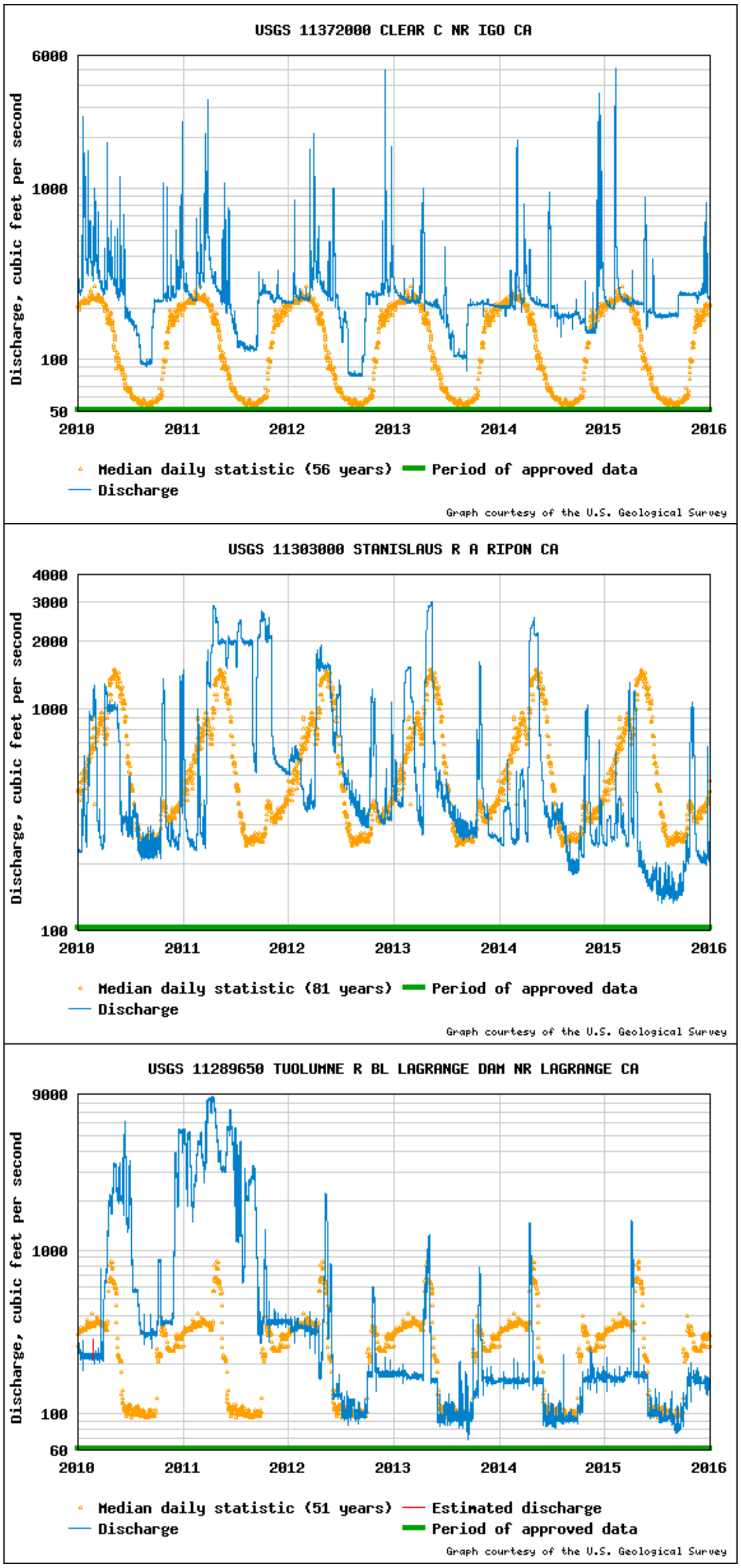

Supplement 2 – Standard deviations of energy expended during holding

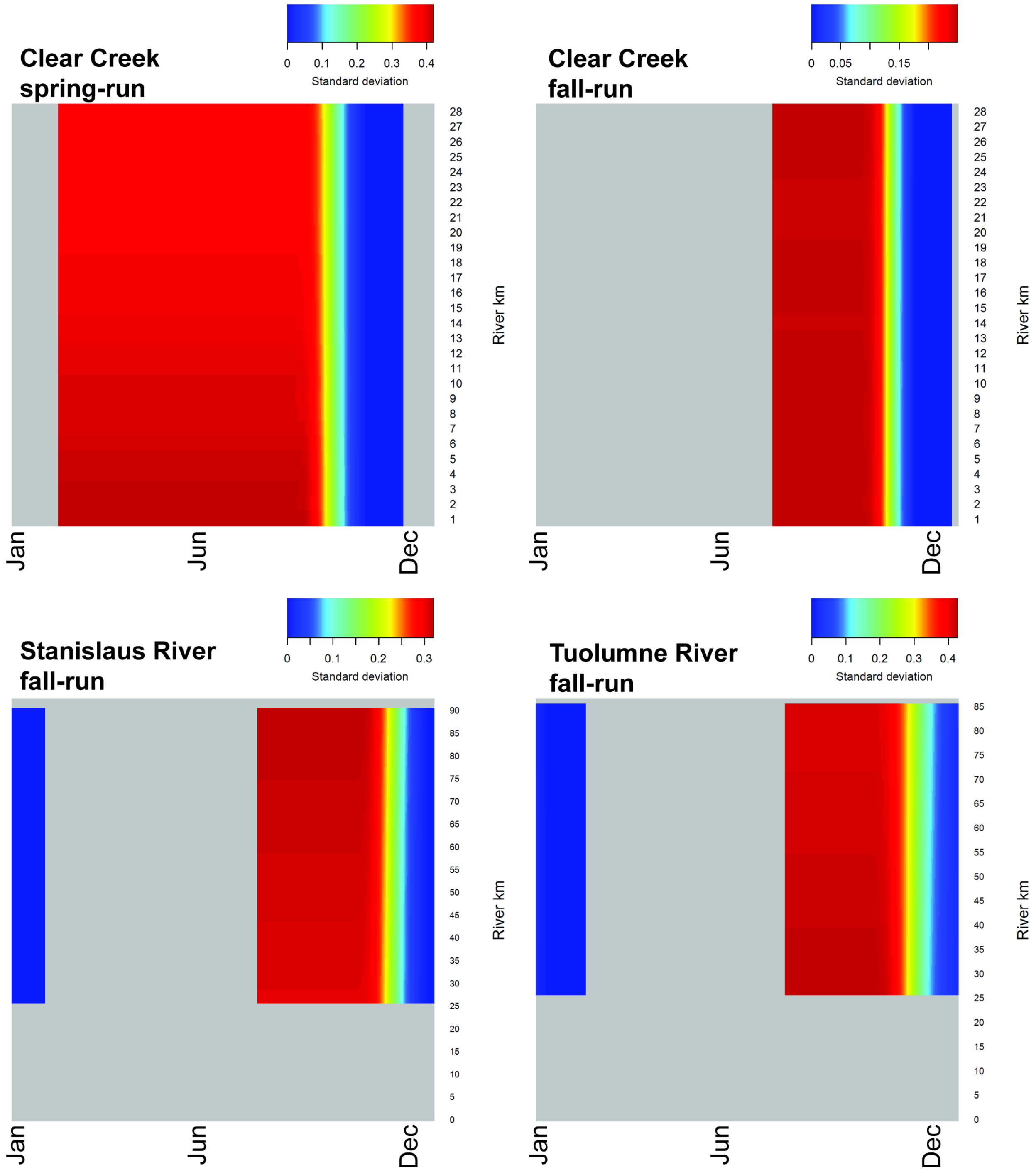

Figure S2.1. Standard deviation of energy expenditure **(E_{hTOT})** of holding Chinook salmon adults from 2013 for spring-run along Clear Creek, fall-run along Clear Creek, fall-run along the Stanislaus River, and fall-run along the Tuolumne River. The model was replicated 1,000 times, sampling from the spawning distribution each time to determine spawn date. The maximum standard deviation was 0.419. Gray locations show spatial-temporal locations where the model was not run (see Methods).

Supplement 3 – Fitting phenological and spatial distribution models

General procedure

Phenology

Survey dates were changed to Julian day and converted into a representative bin size; for example, we used a 14-day bin for surveys completed 1+ times every 2 weeks. Raw counts were converted into percentages based on the total count for that year to weight all years equally regardless of that year's population size. Percentages were rounded to the nearest integer to obtain whole numbers, and observations were multiplied by the integer for a total number of observations of 100*n, where n is the number of years in a dataset, resulting in a relative count per bin size by year. We first graphed each dataset using a histogram and fit a Gaussian (normal) distribution unless otherwise stated. To show goodness of fit, we graphed the empirical and theoretical densities. Once the best fitting distribution was determined, we converted all models into a daily model by multiplying by the bin size.

Spatial distribution

We standardized survey counts to number of observations per 1 river km to match the spatial resolution of the temperature data. In most cases this resulted in the total counts per reach averaged per km. Raw counts were converted into percentages based on the total count for that year to weight all years equally regardless of that year's population size. Percentages were rounded to the nearest integer to obtain whole numbers, and observations were multiplied by the integer for a total number of observations of 100*n, where n is the number of years in a dataset, resulting in a relative count per river km by year (Fig. S3.1).

Phenology

Clear Creek

Arrival time for spring-run Chinook salmon (SCS) was determined from video monitoring near the junction with the Sacramento River. The proportion of annual passage from 2013-2016 was digitized from Clear Creek Technical Team (2017) so we did not have to calculate the frequency distribution from raw counts. For this dataset, frequency was listed by month, so we first fit a normal monthly distribution and then multiplied results by the average numbers of days in a month (30.4375).

For Clear Creek spawning phenology, multiple redd surveys from raw, published, and technical reports were aggregated for the years 2003-2016. A temporary picket weir is installed annually at the boundary of reach 5a/5b (RKM 11.9) to help separate SCS and fall-run Chinook salmon (FCS); occasionally, two weirs are installed (second weir at the boundary of reach 5b/6, RKM 10.5). Prior to weir installation, all fish in the creek are classified as SCS. After installation, all live fish and redds upstream of the picket weir were assigned as SCS, whereas all live fish and redds downstream of the weir were assigned as FCS after installation. Although the Gorge Cascade (demarcating the boundary of reach 5b/6 and the location of the second weir) is a partial barrier to FCS (S. Provins, pers. comm., 2018), peak spawning in reach 5b occurs in early October, whereas 95% of SCS spawning occurs during September (Giovannetti and Brown 2009). Ambiguous live fish or redds in this reach were therefore removed from the dataset. Years with fewer than 10 redds reported (2010, 2017) were also removed. A biweekly normal distribution was fit, and then results were multiplied by 14.

Daily video counts of fall-run arrivals on Clear Creek were obtained from 2012-2016 (Killam and Johnson 2013, Killam et al. 2014, Killam et al. 2015, Killam et al. 2016, Killam et al. 2017). To avoid misclassifications, we removed all arrivals before September 1, by which time the weir is closed and fall-run fish cannot travel further upstream to the spring-run spawning grounds. Arrivals past the last recorded day of new redds were also removed because presumably these fish did not spawn successfully. We fit the dataset to a normal distribution.

An accurate count of fall-run redds along Clear Creek is difficult because of the high density of spawners – sometimes over 8,000 adults in a ~12 km reach (Earley et al. 2013), so we used the distribution of fall spawn timings from nearby Scott River as a proxy. Although Scott River is not classified as a Central Valley population, spawn timing seems similar, peaking in mid-October or early November (Meneks 2017, 2018). In contrast, fall-run in the San Joaquin begin spawning in early November, peak in December, and can spawn into early January (Castle et al. 2016). Along Scott River, surveys were run 1+ times per week, so we used a 7-day bin to fit the spawning distribution. We used 2017 data only because the surveys in 2016 appeared to miss the beginning of spawning (Meneks 2017, 2018).

Stanislaus River

Observations of fall-run passing the weir (~RKM 52.1) on the Stanislaus River were obtained for 2003-2018 (S. Tsao and G. Murphy, pers. comm., 2018). Observations are from the VAKI weir monitoring system, which uses an infrared camera to take motion-triggered photographs when fish pass the weir (J.D. Wikert, pers. comm., 2018). Although salmon were observed swimming upstream year-round, two peaks occurred, a small peak near the end of May and a predominant peak in November. The late-May peak likely represent spring-run Chinook salmon based on arrival timing, although it is currently unclear if spring-run spawn successfully along the Stanislaus (but see Franks 2014). Observations prior to September 1 were not included in the model due to potential misclassification. Observations past the last reported date of spawning were also removed. We fit a normal distribution to weekly count data. The year 2011 had 776 arrivals, but the mean arrival date was ~3 weeks later than average potentially because of high flows in early fall (J.D. Wikert, pers. comm., 2018), so this year was not included when fitting an arrival distribution.

Spawn timing was based on weekly surveys of redds in 2018 (S. Tsao and G. Murphy, pers. comm., 2018). Redd surveys began October 1 and ended mid-January. A total of 3,344 redds were counted in 2018. We fit a normal distribution to the weekly data.

Tuolumne River

VAKI photographic observations of fall-run passing the weir on the Tuolumne River were obtained for 2009-2018 (S. Tsao and G. Murphy, pers. comm., 2018); the weir is located at river km 39.4 (Stillwater Sciences 2013). Chinook salmon passed the weir from mid-September to well into June. However, no new redds were observed after mid-March in 2012-2013 (FISHBIO 2013), so we removed arrivals after mid-April (~day 106) because these late arrivals are likely not spawning successfully. We fit a normal distribution to weekly count data.

Weekly redd counts were obtained for the brood years 2004, 2005, 2007, 2008, and 2012 from published sources and technical reports (Blakeman 2005, 2006, 2008; O'Brien 2009; FISHBIO 2013). We fit a normal distribution to the weekly data.

Spatial distribution

Clear Creek

Spring-run redd surveys are completed ~biweekly by the U.S. FWS Red Bluff Office; we obtained redd locations from 2003-2017 (S. Provins *unpublished*). Redds found downstream of river km 12 (the approximate location of the temporary segregation weir) after September 1 (the weir is set up at the end of August) were removed because they may have been late-arriving spring-run or early spawning fall-run. The years 2010 and 2017 were removed because fewer than 10 redds were reported. The spatial dataset was not normal ($p < 0.001$), and the best fitting distribution was uniform (Fig. S3.1).

Because estimating fall-run redd counts is difficult due to the high density of spawners (Earley et al. 2013), spawning area mapping (SAM) documents the spatial locations and quantity of spawning habitat use (SHU) via counts of redds in October and December. SHU quantities were reported every 1,000 ft reach from 2000-2007 (mean) and annually from 2008-2012 (Earley et al. 2013). We converted reaches to 1 km segments, and used the SHU quantities to fit a normal distribution (Fig. S3.1).

Spring-run juveniles rear throughout the entirety of Clear Creek (U.S. FWS 2015), so we sampled from a uniform distribution from river km 0 (confluence with Sacramento River) to 29 (Whiskeytown Dam). Fall-run juveniles, on the other hand, rear only in the downstream sections of Clear Creek below the Clear Creek Road gravel site (U.S. FWS 2015), so we employed a uniform distribution from river km 0 to 13.5.

Stanislaus River

One year of spatial redd surveys was obtained (S. Tsao and G. Murphy, pers. comm., 2018). These surveys were completed in 2017 for every river mile beginning at Goodwin Dam (RM 58.4; RKM 93.9) to the Stanislaus weir (~RM 32.4; ~RKM 52.1). We fit a Weibull distribution to the spatial redd data, then converted from miles to kilometers.

Juveniles, especially fry, appear to have suitable habitat along the entirety of the accessible sections of the Stanislaus River, from Goodwin Dam downstream to the San Joaquin River; the direct amount of available habitat appears limited by discharge (Bowen et al. 2012). We therefore sampled rearing habitat from a uniform distribution along the entire river.

Tuolumne River

We obtained fall-run redd counts, completed weekly at riffles every river mile, for the brood years 2004, 2005, 2007, 2008, and 2012 (Blakeman 2005, 2006, 2008; O'Brien 2009; FISHBIO 2013). These surveys are conducted with a single pass, and redd superimposition can lead to undercounting, particularly in the upper reaches of the Tuolumne River where most of the spawning occurs. Still, all years found a similar pattern in redd distribution except for 2005 which showed almost as many redds from RM 34-42 as in the upper reaches (Blakeman 2006); we therefore removed year 2005 from our dataset. Because of the potential for undercounting in the upper reaches, we fit a normal distribution using the median (RM = 48) instead of the mean (RM = 44.9). Results were converted to river kilometers to match our interpolated temperature dataset and clipped over RKM 86 because of the LaGrange Dam.

We assumed that rearing could occur uniformly along the Tuolumne River, and therefore sampled rearing habitat from a uniform distribution along the entire river, from the LaGrange Dam to the confluence with the San Joaquin River.

Table S3.1. Summary statistics of the empirical phenological datasets used to estimate arrival time and spawning time for each river. Weighted thermal effects were calculated by sampling from each fitted distribution (see Supplement 1 for phenological distribution fitting). Values are in Julian day.

Population	Mean	SD	Median	Min	Max	Skewness	Kurtosis
Clear Creek spring-run arrival	164	27	152	91	243	0.25	3.83
Clear Creek spring-run redd	276	11	280	238	322	0.73	4.5
Clear Creek fall-run arrival	289	15	288	245^	349*	0.23	3.10
Clear Creek fall-run redd`	307	8	308	287	329	-0.10	3.08
Stanislaus River fall-run arrival	308	18	308	259^	350*	0.08	2.76
Stanislaus River fall-run redd	331	11	329	287	350	-0.06	2.28
Tuolumne River fall-run arrival	316	23	315	259	76	1.02	5.65
Tuolumne River fall-run redd	329	14	329	280	76	0.41	4.41

`note that spawning phenology based on nearby Scott River
^earlier observations removed due to potential misidentification
*later observations removed because no new redds observed

Table S3.2. Summary statistics of the empirical spatial datasets used to estimate spawning distribution and juvenile rearing distribution. Weighted thermal effects were calculated by sampling from each fitted distribution (see Supplement 1 for spatial distribution fitting).Values are in river kilometer, where '0' is the river mouth.

Population	Mean	SD	Median	Min	Max	Skewness	Kurtosis
Clear Creek spring-run redd	20.83	5.12	20.5	12.5	29.5	0.057	1.59
Clear Creek fall-run redd	7.98	1.63	8.5	3.5	10.5	-0.635	2.89
Clear Creek spring-run rearing^	NA	NA	NA	0	29.5	NA	NA
Clear Creek fall-run rearing^	NA	NA	NA	0	13.5	NA	NA
Stanislaus River fall-run redd	76.1	10.6	77.9	52.1	93.9	-0.675	2.47
Stanislaus River fall-run rearing^	NA	NA	NA	0	93.9	NA	NA
Tuolumne River fall-run redd	72.30	9.75	77.23	38.6	83.7	-1.003	3.01
Tuolumne River fall-run rearing^	NA	NA	NA	0	86	NA	NA

^based on description only, not quantified data

Phenology – Arrival and spawning

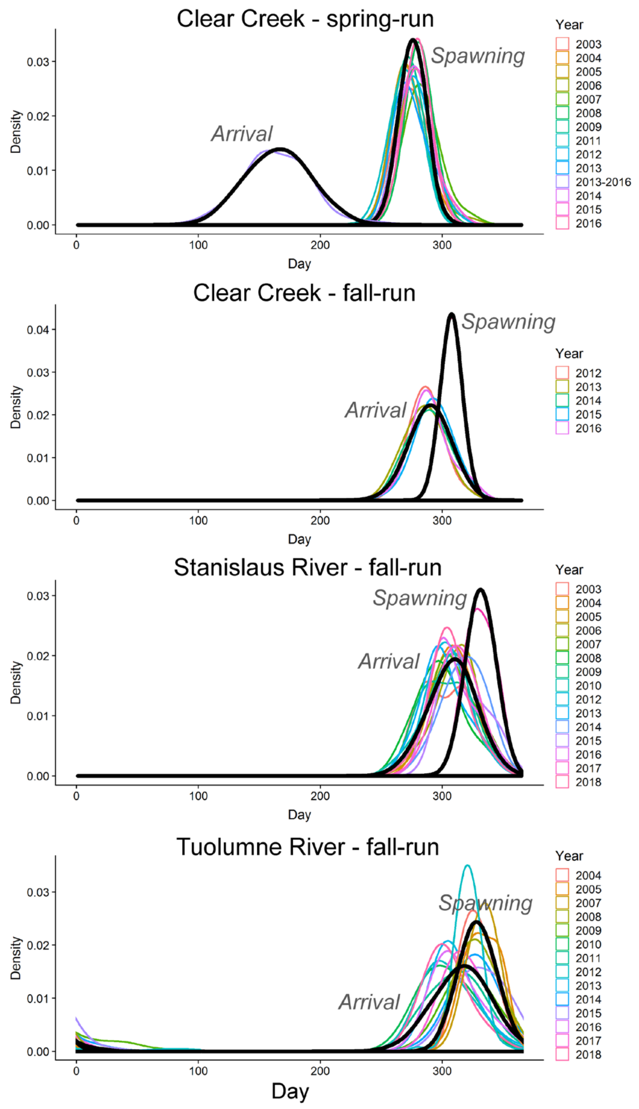

Spatial distribution – Redds

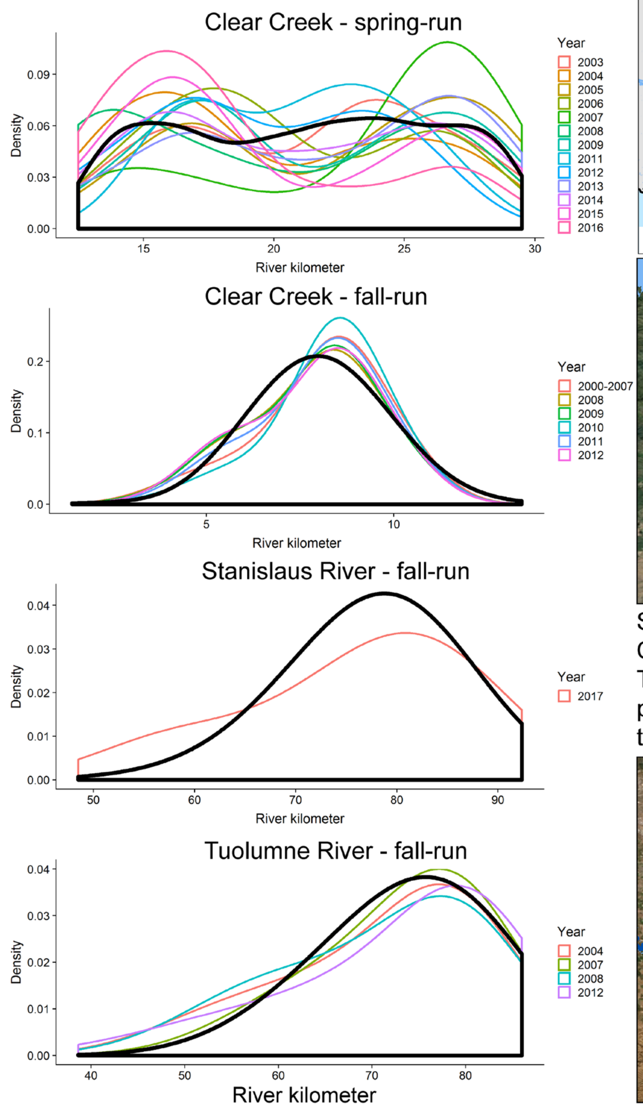

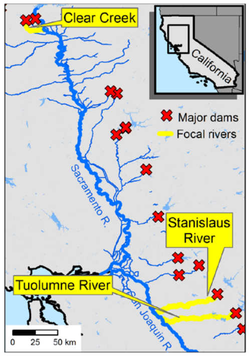

Left: Focal managed rivers for this study. Clear Creek includes both spring- and fall-run. Stanislaus and Tuolumne Rivers have only fall-run.

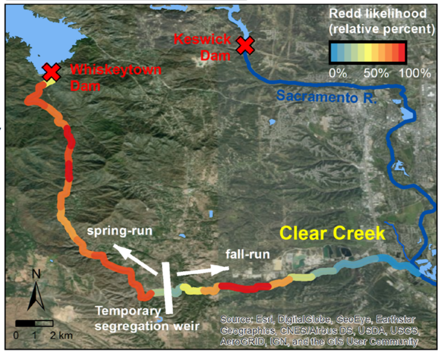

Spatial distribution of redds along Clear Creek (above) and Stanislaus and Tuolumne Rivers (below). Likelihood is percentage of highest redd count along the river; runs were counted separately.

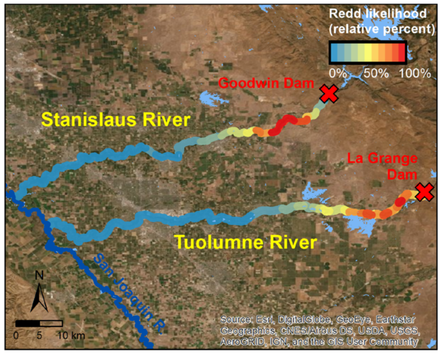

Figure S3.1. Yearly (colored) and averaged (bold black) distributions of arrival and spawning phenology (left column) and spawning spatial distributions (right column) of spring-run on Clear Creek and fall-runs on Clear Creek, Stanislaus River, and Tuolumne River. In the left column, the earlier peaks are arrivals, and the later peaks are spawning (indicated on panels). The phenology x-axis (left column) shows Julian day. The spatial distribution x-axis (right column) shows the spatial location (river kilometer) of redds. River km 0 is the confluence with the Sacramento River (Clear Creek) or San Joaquin River (Stanislaus and Tuolumne Rivers). Anadromous fish are blocked upstream by major dams: Clear Creek is blocked by the Whiskeytown Dam at RKM 29; the Stanislaus River is blocked by the Goodwin Dam at RKM 94; the Tuolumne River is blocked by the La Grange Dam at RKM 86.

Supplemental References

Blakeman, D. (2005). 2004 Tuolumne River Fall Chinook Salmon Escapement Survey. California Dept. of Fish and Game.

Blakeman, D. (2006). 2005 Tuolumne River Fall Chinook Salmon Escapement Survey. California Dept. of Fish and Game.

Blakeman, D. (2008). 2007 Tuolumne River Fall Chinook Salmon Escapement Survey. Tuolumne River Restoration Center, La Grange Field Office, California Dept. of Fish and Game.

Bowen, M. D., Gard, M., Hilldale, R., Zehfuss, K., & R. Sutton. (2012). Stanislaus River Discharge-Habitat Relationships for Rearing Salmonids. *U.S. Dept. Interior, Bureau of Reclamation, Central California Area Office, Folsom, CA*.

Castle, C., N. Cullen, J. Goodell, Z. Jackson, A. Shriver, M. Workman, and J. Kirsch. (2016). Fall-run Chinook Salmon spawning assessment during 2013 and 2014 within the San Joaquin River, California. *Annual Technical Report, United States Fish and Wildlife Service, Lodi, CA*.

Clear Creek Technical Team. (2016). Annual Report for the Coordinated Long-Term Operation Biological Opinion. *Clear Creek Restoration Program, Central Valley Project Improvement Act.*

Clear Creek Technical Team. (2017). Annual Report for the Coordinated Long-Term Operation Biological Opinion. *Clear Creek Restoration Program, Central Valley Project Improvement Act.*

Earley, J. T., D. J. Colby, & M. R. Brown. (2013). Juvenile salmonid monitoring in Clear Creek, California, from October 2010 through September 2011. U.S. Fish and Wildlife Service, Red Bluff Fish and Wildlife Office, Red Bluff, California.

FISHBIO. (2013). Salmonid redd mapping study report, Don Pedro Project. FERC No. 2299. Turlock Irrigation District, Turlock, CA, & Modesto Irrigation District, Modesto, CA.

Franks, S. (2014). Possibility of natural producing spring-run Chinook salmon in the Stanislaus and Tuolumne rivers. *Unpublished Work. National Oceanic Atmospheric Administration*.

Giovannetti, S.L. & Brown, M.R. (2009). Adult spring Chinook salmon monitoring in Clear Creek, California. *2008 annual report, U.S. Fish and Wildlife Service, Red Bluff Fish and Wildlife Office, Red Bluff, California*.

Killam, D. & Johnson, M. (2013). Chinook Salmon Populations of the Upper Sacramento River Basin In 2012. *California Department of Fish and Wildlife-Northern Region, Red Bluff Fisheries Office, RBFO Technical Report No. 02-2013.*

Killam, D., Johnson, M., & Revnak, R. (2014). Chinook Salmon Populations of the Upper Sacramento River Basin In 2013. *California Department of Fish and Wildlife-Northern Region, Red Bluff Fisheries Office, RBFO Technical Report No. 02-2014*.

Killam, D., Johnson, M., & Revnak, R. (2015). Chinook Salmon Populations of the Upper Sacramento River Basin In 2014. *California Department of Fish and Wildlife-Northern Region, Red Bluff Fisheries Office, RBFO Technical Report No. 03-2015*.

Killam, D., Johnson, M., & Revnak, R. (2016). Chinook Salmon Populations of the Upper Sacramento River Basin In 2015. *California Department of Fish and Wildlife-Northern Region, Red Bluff Fisheries Office, RBFO Technical Report No. 03-2016*.

Killam, D., Johnson, M., & Revnak, R. (2017). Salmonid Populations of the Upper Sacramento River Basin In 2016. *California Department of Fish and Wildlife-Northern Region, Red Bluff Fisheries Office, RBFO Technical Report No. 03-2017*.

Meneks, M. (2017). 2016 Fall Chinook Salmon Spawning Ground Survey. *Salmon-Scott Rivers Ranger District, Klamath National Forest*.

Meneks, M. (2018). 2017 Fall Chinook Salmon Spawning Ground Survey. *Salmon-Scott Rivers Ranger District, Klamath National Forest*.

Provins, S. (*unpublished*). Temperature and Discharge Dependent Mortality of Juvenile Spring-Run Chinook Salmon in Clear Creek. *U.S. Fish & Wildlife Service, Red Bluff Office*.

O'Brien, J. (2009). 2008 Tuolumne River Fall Chinook Salmon Escapement Survey. Tuolumne River Restoration Center, La Grange Field Office, California Dept. of Fish and Game.

Stillwater Sciences. (2013). Chinook salmon population model study. Don Pedro Project, FERC No. 2299. Turlock Irrigation District, Turlock, CA, & Modesto Irrigation District, Modesto, CA.

U.S. Fish & Wildlife Service [U.S. FWS]. (2015). Clear Creek habitat synthesis report. *Anadromous Fish Restoration Program. Report to Central Valley Project Improvement Act (CVPIA) Instream Flow Investigations.*
